# PARP2 Is the Predominant Poly(ADP-Ribose) Polymerase in Arabidopsis DNA Damage and Immune Responses

**DOI:** 10.1371/journal.pgen.1005200

**Published:** 2015-05-07

**Authors:** Junqi Song, Brian D. Keppler, Robert R. Wise, Andrew F. Bent

**Affiliations:** 1 Department of Plant Pathology, University of Wisconsin - Madison, Madison, Wisconsin, United States of America; 2 Department of Biology, University of Wisconsin - Oshkosh, Oshkosh, Wisconsin, United States of America; Virginia Tech, UNITED STATES

## Abstract

Poly (ADP-ribose) polymerases (PARPs) catalyze the transfer of multiple poly(ADP-ribose) units onto target proteins. Poly(ADP-ribosyl)ation plays a crucial role in a variety of cellular processes including, most prominently, auto-activation of PARP at sites of DNA breaks to activate DNA repair processes. In humans, PARP1 (the founding and most characterized member of the PARP family) accounts for more than 90% of overall cellular PARP activity in response to DNA damage. We have found that, in contrast with animals, in *Arabidopsis thaliana* PARP2 (At4g02390), rather than PARP1 (At2g31320), makes the greatest contribution to PARP activity and organismal viability in response to genotoxic stresses caused by bleomycin, mitomycin C or gamma-radiation. Plant PARP2 proteins carry SAP DNA binding motifs rather than the zinc finger domains common in plant and animal PARP1 proteins. PARP2 also makes stronger contributions than PARP1 to plant immune responses including restriction of pathogenic *Pseudomonas syringae* pv. *tomato* growth and reduction of infection-associated DNA double-strand break abundance. For poly(ADP-ribose) glycohydrolase (PARG) enzymes, we find that Arabidopsis PARG1 and not PARG2 is the major contributor to poly(ADP-ribose) removal from acceptor proteins. The activity or abundance of PARP2 is influenced by PARP1 and PARG1. PARP2 and PARP1 physically interact with each other, and with PARG1 and PARG2, suggesting relatively direct regulatory interactions among these mediators of the balance of poly(ADP-ribosyl)ation. As with plant PARP2, plant PARG proteins are also structurally distinct from their animal counterparts. Hence core aspects of plant poly(ADP-ribosyl)ation are mediated by substantially different enzymes than in animals, suggesting the likelihood of substantial differences in regulation.

## Author Summary

All living organisms face constant challenges from environmental factors. Appropriate and rapid responses to external stimuli are crucial for maintenance of genome integrity and cell survival. Poly(ADP-ribosyl)ation is a post-translational modification and contributes to multiple molecular and cellular processes including a prominent role in DNA damage repair. Human PARP1, the founding and most characterized member of the PARP family, accounts for more than 90% of overall molecular and cellular PARP activity in response to DNA damage while PARP2 supplies a minor portion of this PARP activity. Here we show that Arabidopsis PARP2 rather than PARP1 plays the predominant role in poly(ADP-ribosyl)ation and organismal resilience in response to either chemically-induced DNA damage or pathogen infections. We show that the activity and abundance of PARP2 is regulated by both PARP1 and PARG1. We also show that Arabidopsis PARG1 rather than PARG2 is the major contributor to removal poly(ADP-ribose) from acceptor proteins. Core aspects of plant poly(ADP-ribosyl)ation are mediated by substantially different enzymes than in animals, suggesting the likelihood of substantial differences in regulation.

## Introduction

Appropriate and rapid responses to external stimuli can be crucial for maintenance of cellular and organismal viability, especially under stress conditions. Both biotic and abiotic stresses can induce genome DNA damage [1–4]. Maintenance of genome integrity via DNA damage repair then becomes essential, in both germ-line and somatic cells [2, 5, 6].

Poly(ADP-ribosyl)ation is a post-translational modification mediated by poly(ADP-ribose) polymerase (PARP) enzymes, in which negatively charged ADP-ribose units are transferred from donor nicotinamide adenine dinucleotide (NAD^+^) molecules onto target proteins [7]. PARP enzymes are themselves the most prominent poly(ADP-ribosyl)ation target. Poly(ADP-ribosyl)ation plays a key role in a wide range of cellular responses including DNA repair, chromatin modification, control of transcription and cell death [7–9]. Poly(ADP-ribosyl)ation and PARP proteins have been identified in a wide variety of plants and animals as well as bacteria, fungi and double-stranded DNA viruses [10–12]. In humans, 17 PARP proteins have been identified based on homology to PARP1, the founding member of the PARP family [13]. PARP1 accounts for approximately 90% of the PARP activity in mammalian cells under genotoxic situations, while PARP2 is apparently responsible for the remaining 10% [14–16].

The Arabidopsis genome encodes three PARP proteins that carry a PARP signature motif, as well as RCD1 and five SRO (“Similar to RCD One”) proteins with a variant form of the PARP signature [11, 17–19]. Although the names of plant PARP proteins have in some instances been reversed, the product of the Arabidopsis *At2g31320* gene (NCBI NP_850165.1) is herein called PARP1 and the Arabidopsis *At4g02390* product (NCBI NP_192148.2) is PARP2, based on their relative similarities to the earlier-named and extensively studied animal homologs (S1 Fig) [11, 20]. PARP2-like proteins are broadly conserved across diverse plant taxa (S2 Fig), while PARP1 is broadly conserved across plants and animals [11, 18]. Arabidopsis PARP3 contains variant active site residues that suggest lack of PARP catalytic function [12], and expression of Arabidopsis PARP3 is restricted to seed tissues [21]. The SROs (including RCD1) are a conserved family of plant-specific proteins that have functions in development and abiotic stress responses [19]. Although a wheat SRO protein that does possesses PARP activity was recently described [22], Arabidopsis SROs contain variant PARP motifs that both bioinformatically and biochemically were found to lack ADP-ribosyl transferase activity [19].

PARPs are widely known for their roles in genotoxic stress, DNA damage repair and programmed cell death in animals [13, 23, 24]. Although additional roles for PARP enzymes are being discovered [9], one of the best-known roles of PARPs is their function as DNA damage sensors. PARP1 in particular binds in its poly(ADP-ribosyl)ated form to ssDNA and dsDNA breaks and initiates events that attract DNA damage repair machinery to the sites of damage [7, 25]. A growing body of evidence indicates that plant PARPs have similar functions. PARP proteins are involved in microhomology mediated back-up non-homologous end joining in Arabidopsis [20]. Arabidopsis PARP1 and PARP2 accumulate rapidly and strongly in response to ionizing radiation, whereas PARP2 is preferentially induced by dehydration and excess cadmium [26]. PARP in soybean cells is differentially involved in responses to mild and intense oxidative stresses, through regulating DNA repair and programmed cell death [27]. Knocking down PARP activities in Arabidopsis and oilseed rape plants by chemical inhibition or gene silencing inhibited cell death and made plants more tolerant to a broad range of abiotic stresses including high light, drought and heat [28, 29]. This is due at least in part to induction of specific abscisic acid signaling pathways [29]. PARP inhibition also enhances Arabidopsis growth by promoting the leaf cell number [30, 31]. In animals, PARP proteins have been implicated in regulation of telomere length, telomere activity and chromosome end protection [32, 33]. However, it appears that Arabidopsis PARP proteins make limited contributions to telomere regulation and maintenance, although telomere dysfunction triggers PARP activation [34].

Poly(ADP-ribosyl)ation is reversible; covalently attached poly(ADP-ribose) can be cleaved from acceptor proteins by poly(ADP-ribose) glycohydrolase (PARG) [7, 8]. Mammalian genomes encode a single *PARG* gene [35, 36] and mutation of *PARG* caused enhanced sensitivity to genotoxic stress and elevated accumulation of poly(ADP-ribose), leading to embryonic lethality in mice and *Drosophila* [37, 38]. However, the human *PARG* gene undergoes alternative splicing, resulting in multiple PARG protein isoforms that localize to different cellular compartments [39]. Unlike animal models the Arabidopsis genome encodes two *PARG* genes, *PARG1* and *PARG2*, with 52% amino acid identity (S1 Fig) [11]. *PARG1* and *PARG2*, as well as an inactive pseudogene *At2g31860*, are all located adjacent to each other on Arabidopsis chromosome 2 [11]. Much less is known about the function of PARGs than PARPs in plant poly(ADP-ribosyl)ation. PARG1 was originally identified to play a role in circadian oscillation in Arabidopsis [40]. The Arabidopsis *PARG2* gene is robustly induced by pathogen-associated molecular patterns (PAMPs) and numerous different pathogens, but disruption of *PARG1*, not *PARG2*, altered various plant defense responses [17, 41]. Similar to its counterpart, PARP, Arabidopsis PARG1 has also been implicated in drought, osmotic and oxidative stress responses [42].

Despite the above work, the mechanisms by which PARPs and PARGs regulate diverse cellular processes in plants remain largely unknown. The present study used mutational and biochemical approaches to assess the relative contributions of Arabidopsis PARP1/2 and PARG1/2. We present evidence that, unlike in animals, PARP2 rather than PARP1 plays the major role in plant DNA damage and immune responses. We also demonstrate that PARG1 rather than PARG2 is the primary enzyme that counteracts poly(ADP-ribosyl)ation in Arabidopsis. In addition, we discover that PARP1 associates with PARP2, and that PARP1 and PARP2 interact with both PARG1 and PARG2.

## Results

### Mutant *parp2* plants are more sensitive to DNA damage agents than wild-type or *parp1* plants

**Fig 1:**
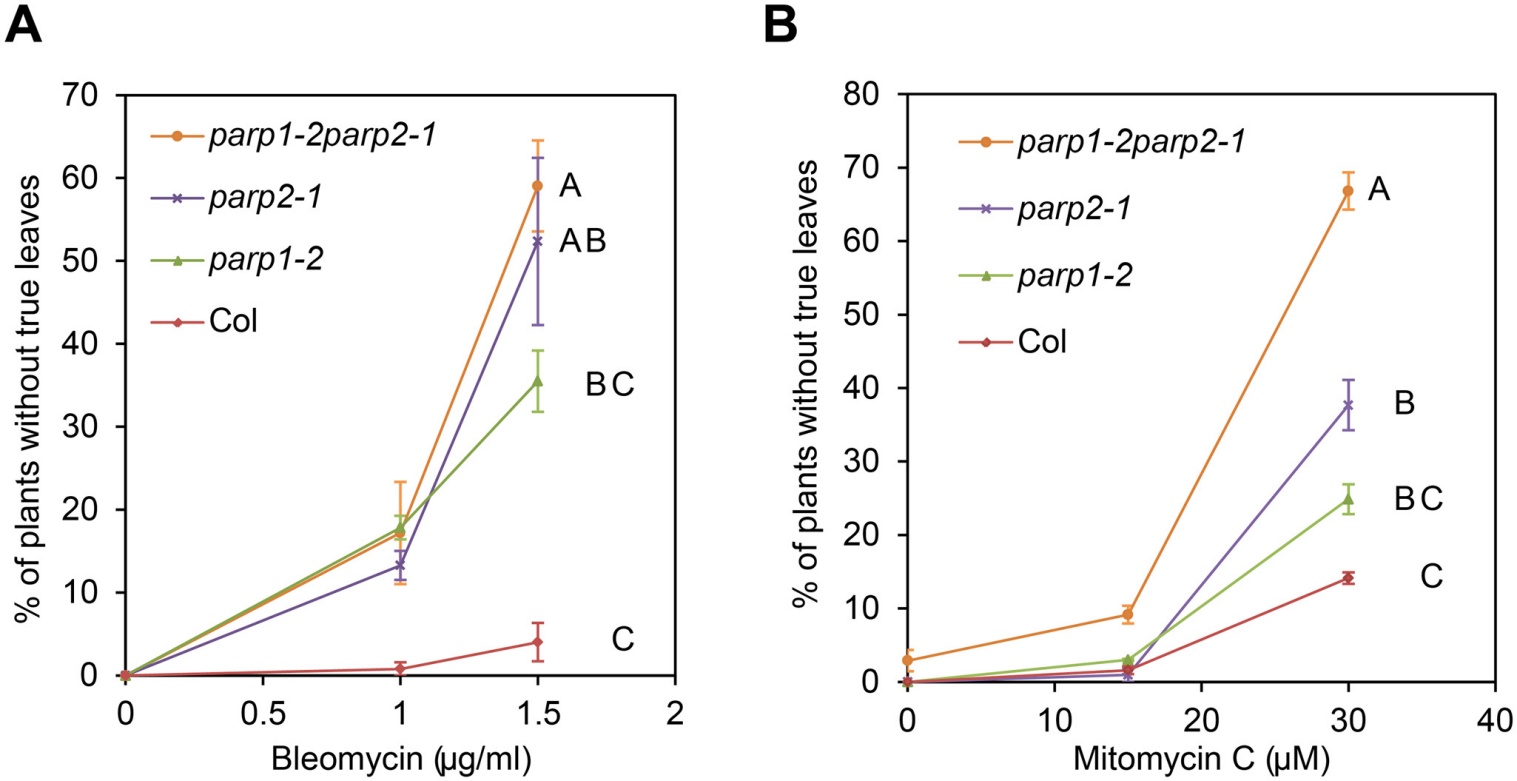
Arabidopsis *parp* mutants are hypersensitive to DNA damage agents. Wild-type Col-0 and *parp* mutant seeds were grown on MS agar medium supplemented with the genotoxic agents bleomycin (A) or mitomycin C (B). Sensitivity to DNA damage agents was scored as the percentage of plants that had not yet developed true leaves after 14 d. Mean and standard error of the mean are shown for one experiment; experiment was performed three times with similar results. Genotypes not sharing same letter on graph are significantly different at the high concentration (ANOVA Tukey HSD *P* < 0.05 across three experiments).

To examine the functional importance of PARP1 and PARP2 in plant poly(ADP-ribosyl)ation and in effective responses to DNA damage agents, we tested sensitivity to genotoxic agents in Arabidopsis *parp1* and *parp2* single mutants and in *parp1parp2* double mutants. Two Arabidopsis T-DNA insertion lines with mutations in PARP1 (At2g31320) and one for PARP2 (At4g02390) were identified: *parp1-1* (GABI_380E06), *parp1-2* (GABI_382F01) and *parp2-1* (GABI_420G03). Double mutant *parp1parp2* plants were generated by genetic crosses. The chemical bleomycin is a potent inducer of DNA double-strand breaks (DSB), with a mode of action similar to that of ionizing radiation [43]. Plants were grown on MS plates supplemented with bleomycin and organismal-level sensitivity to DNA damage was scored as the number of plants without true leaves 14 days after germination [44, 45]. As shown in Fig 1A, in bleomycin-free MS plates almost all wild-type and mutant plants produced normal true leaves. In MS plates supplemented with 1.5 μg/ml of bleomycin, over 95% of wild-type plants still produced true leaves, whereas approximately 35% of the *parp1-2* plants had no true leaves. Strikingly, more than 50% of the *parp2-1* single mutants failed to generate true leaves, similar to *parp1-2parp2-1* double mutants (Fig 1A). RT-PCR analysis confirmed that expression of *PARP1* or *PARP2* was abolished in the respective mutants (S3 Fig). Although contributions of PARP1 cannot be ruled out, ANOVA across three replicate experiments indicated that the growth defects on bleomycin were significantly worse than wild-type only for the *parp2* and *parp1parp2* double mutants. The experiments suggest that PARP2 is a more substantial contributor than PARP1 to a successful response to bleomycin. Similarly, increased sensitivity to the DNA alkylating agent methyl methane sulfonate (MMS) has been observed in a *parp1parp2* double mutant [20, 34].

To further examine the role of PARP1 and PARP2 in DNA damage repair in Arabidopsis we analyzed sensitivity to mitomycin C, an interstrand DNA crosslinking agent [46]. Similar to bleomycin treatment, the *parp1-2* and *parp2-1* mutants exhibited moderately or markedly increased sensitivity to mitomycin C, respectively (Fig 1B). ANOVA across three replicate experiments again indicated that the growth defects, this time in response to mitomycin C, were significantly worse than wild-type only for the *parp2* and *parp1parp2* double mutants. However, the *P*-value for the Col vs. *parp1* contrast was 0.054 (very close to *P* < 0.05 significance), and PARP1 contributions were also indicated because the *parp1parp2* double mutant grew significantly less well than the *parp2* single mutant. Similar results after mitomycin C treatment also were obtained using a second *parp1* mutant allele and *parp1/parp2* double mutant line (S4 Fig). These mitomycin C experiments indicate that PARP1 and PARP2 are both required for effective repair of damaged DNA, but that PARP2 plays a stronger role in tolerance of plant DNA damage.

### PARP2 accounts for most of the DNA damage-induced PARP activity in Arabidopsis

**Fig 2:**
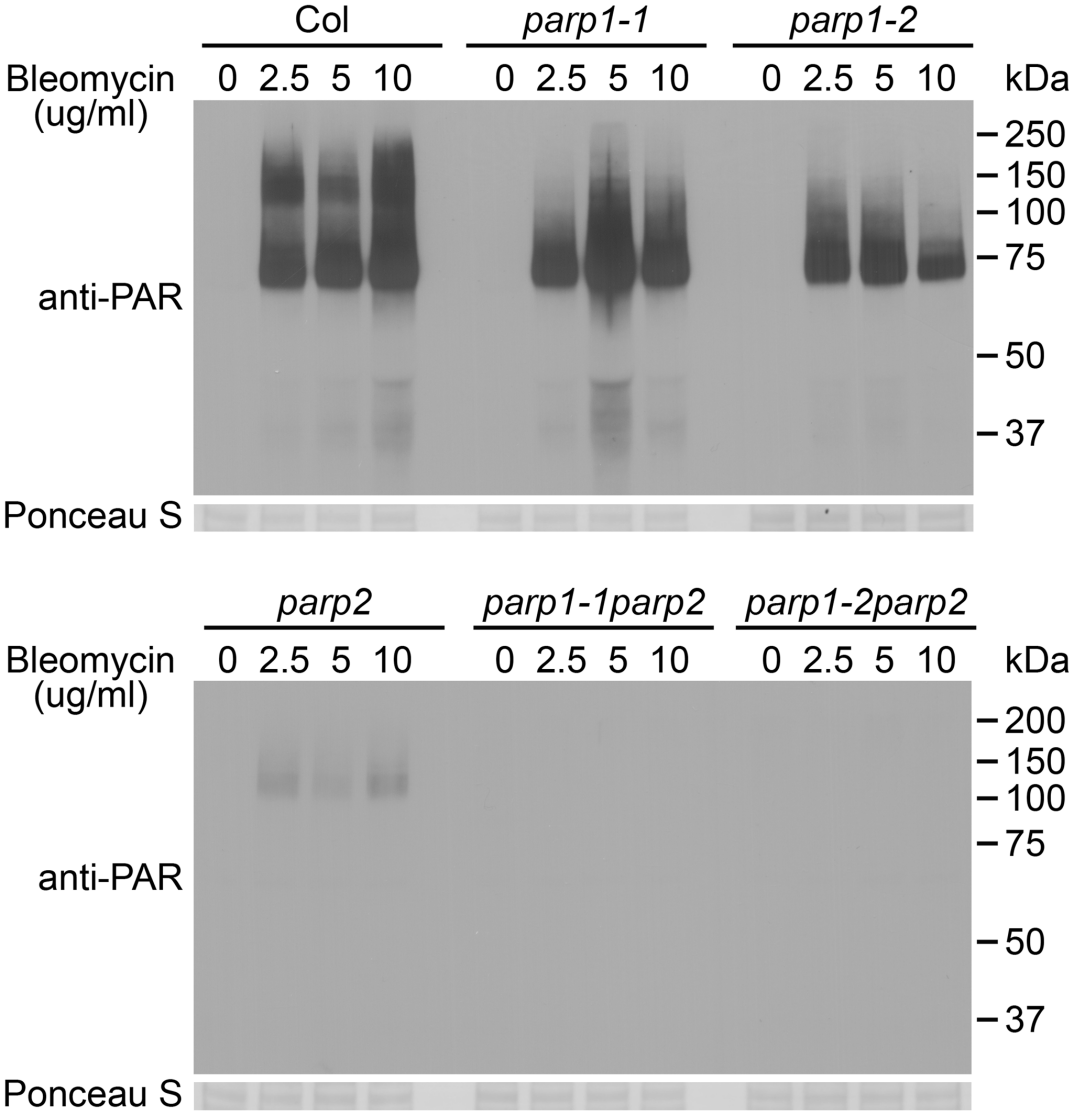
PARP2 plays a dominant role in DNA damage response after bleomycin treatment. Two-week old *Arabidopsis* plants were transferred to 0, 2.5, 5 or 10 μg/ml of bleomycin for 18 h. Total proteins were extracted, separated by SDS-PAGE and analyzed by immunoblotting using an anti-PAR antibody. Equivalent loading of lanes was verified using Ponceau S stain. All samples shown and both blots were processed in parallel within the same experiment. Similar results were obtained in three separate experiments.

To investigate whether disruption of the Arabidopsis *PARP1* or *PARP2* genes disrupts *in planta* poly(ADP-ribosyl)ation of target proteins, wild-type and *parp* mutant plants were treated for 18h with increasing concentrations of bleomycin and poly(ADP-ribosyl)ated proteins were monitored on protein immunoblots using an anti-PAR (anti-poly(ADP-ribose)) antibody (Fig 2). Greatly increased levels of poly(ADP-ribosyl)ation were observed in wild-type plants after treatment with bleomycin at concentrations ranging from 2.5 to 10 μg/ml. Substantial amounts of poly(ADP-ribosyl)ated proteins were still detected despite knockout of PARP1 in the *parp1-1* or *parp1-2* single mutants. This is unexpected given that Arabidopsis PARP1 is the zinc finger-containing homolog of animal PARP1, which has been abundantly demonstrated to make the greatest contribution to poly(ADP-ribosyl)ation in response to DNA damage [7, 10, 14–16]. Knockout of PARP2 rather than PARP1 severely depleted detectable PARP activity in Arabidopsis, with only marginal elevation of poly(ADP-ribosyl)ated proteins detected after bleomycin treatment of *parp2-1* mutant plants (Fig 2). Little or no PARP activity was observed in the *parp1-1parp2-1* or *parp1-2parp2-1* mutants (Fig 2). Under long exposure conditions residual PARP activity in the *parp1-2parp2-1* double mutants still remained barely detectable.

We had obtained RT-PCR evidence that the *parp2-1* mutation eliminated PARP2 transcript (Fig 1B) but further tests were then conducted to confirm loss of PARP2 protein production in the *parp2-1* mutant, as well as to confirm the specificity of a custom-raised PARP2 polyclonal antibody. In wild-type Col-0 plants treated with bleomycin, PARP2 protein was detected using the anti-PARP2 antibody (S5 Fig). PARP2 protein was still detected in *parp1-1* and in *parp1-2* mutant plants, but not in the *parp2-1* line or in two separate *parp1parp2* double mutant lines, indicating that *parp2-1* is a null mutant (S5 Fig).

To confirm that the compromised PARP activity in *parp2-1* is due to the T-DNA insertion in the *PARP2* gene, we complemented the *parp2-1* mutant with a construct carrying native Arabidopsis *PARP2* promoter sequences driving expression of Arabidopsis *PARP2* genomic DNA fused to an HA epitope tag. Five independent T2 transgenic lines with different expression levels of PARP2-HA were chosen for complementation analysis. As shown in S6 Fig, poly(ADP-ribosyl)ation activity was restored in the complemented lines. We further observed that the abundance of poly(ADP-ribosyl)ated proteins present in response to the DNA damaging agent bleomycin correlated with the levels of PARP2 protein in the selected lines (S6 Fig). As a side-matter, the anti-PARP2 antibody was used to detect PARP2 in these complementation experiments. We were not able to detect the PARP2-HA fusion protein using an anti-HA antibody (that worked well with other HA-tagged proteins), possibly due to cleavage or inaccessibility of the C-terminal tag on PARP2. Overall, the above results indicate that PARP1 and PARP2 both contribute to poly(ADP-ribosyl)ation, but that PARP2 is the primary Arabidopsis enzyme responsible for poly(ADP-ribosyl)ation activity in response to DNA damage.

**Fig 3:**
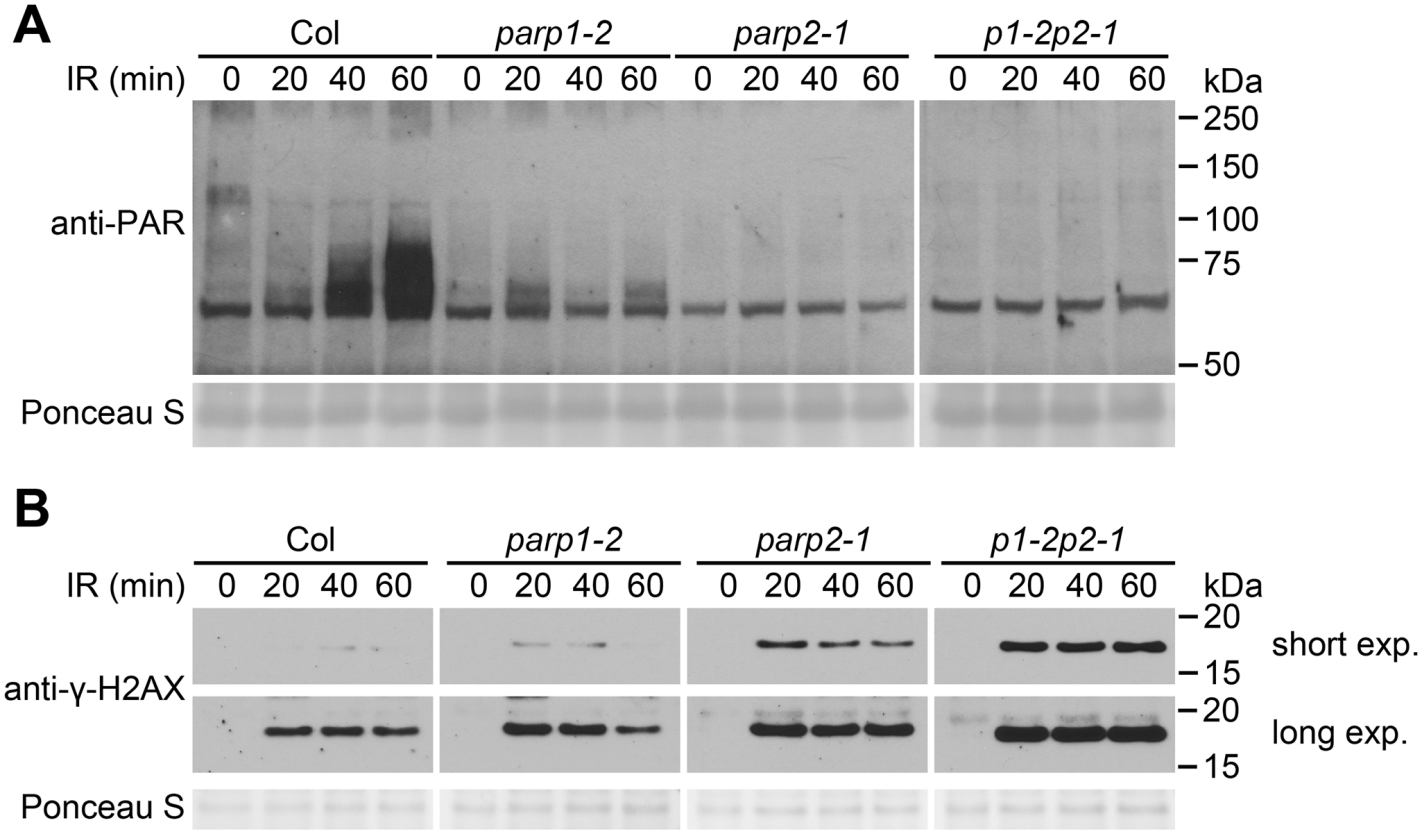
PARP2 plays a dominant role in response to ionizing irradiation. (A) Two-week old Arabidopsis plants grown on MS plates were irradiated with 150 Gy of γ-radiation and then flash-frozen 20, 40 or 60 min after removal from the radiation source. Total proteins were then extracted, separated by SDS-PAGE and analyzed by immunoblotting with an anti-PAR antibody. 0 min sample not exposed to γ-radiation source. All samples shown in (A) were processed in parallel within the same experiment. (B) The level of γ-H2AX was assessed at 20, 40 and 60 min after irradiation as in (A), using an anti-γ-H2AX antibody. Samples all processed in parallel from same experiment. Shorter and longer time exposures of same immunoblot are shown. Equivalent loading of lanes was verified using Ponceau S stain. Experiments were performed three times with similar results.

In related experiments we irradiated plants with 150 Gy of γ-radiation, a dose that is sufficient to induce DNA double strand breaks [47], and PARP activity was monitored (Fig 3A). Markedly increased amounts of poly(ADP-ribosyl)ated proteins were detected in wild-type plants from 20 to 60 min after irradiation. This γ-ray-induced PARP activity was substantially reduced in the *parp1-2* mutant. An even more complete reduction in poly(ADP-ribosyl)ation was observed in the *parp2-1* single mutant, and a similarly complete reduction was observed in the *parp1-2parp2-1* double mutant. With the γ-ray-treated plant samples we also measured the level of phosphorylated histone γ-H2AX, a standard indicator of DNA double-strand breaks [48, 49]. Compared to wild-type plants, in the *parp1-2* single mutants elevated DNA damage was detected as an increase in the intensity of the γ-H2AX band (Fig 3B). More DNA damage was reproducibly detected in the *parp2-1* single mutant than the *parp1-2* mutant, and even more significantly increased DNA damage was observed in the *parp1-2parp2-1* double mutant (Fig 3B). Hence as in earlier experiments where plant growth in the face of DNA damage was monitored (Fig 1), experiments monitoring abundance of γ-ray-induced DNA double-strand breaks (Fig 3B) detected contributions of PARP1 as well as PARP2. However, these experiments with γ-ray-treated plants again indicated that PARP2 accounts for majority of the cellular PARP activity that is activated in response to DNA damage (Fig 3A).

### Subcellular localization of PARP1/2 and PARG1/2 proteins

**Fig 4:**
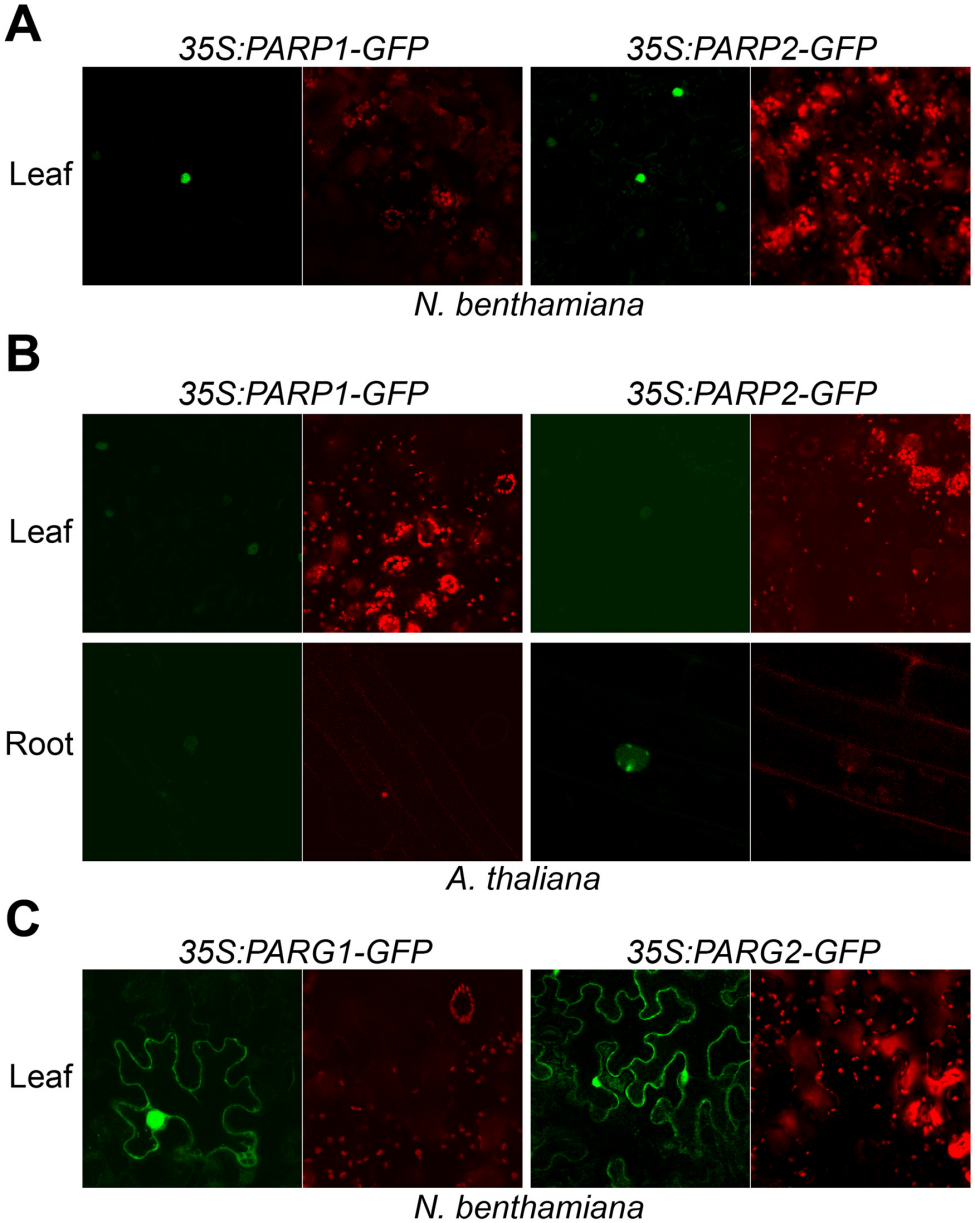
Subcellular localization of Arabidopsis PARP1/2 and PARG1/2. Paired confocal fluorescence microscopy images show same sample; green wavelengths (GFP) on left and red wavelengths (chlorophyll) on right. (A) Arabidopsis PARP1 and PARP2 localized in the nucleus. *35S*:*AtPARP1-GFP* and *35S*:*AtPARP2-GFP* transiently expressed in *N*. *benthamiana* epidermal cells within leaves were imaged. (B) PARP1 and PARP2 localized in the nucleus in Arabidopsis. Subcellular localization was carried out in stable transgenic lines carrying *35S*:*AtPARP1-GFP* and *35S*:*AtPARP2-GFP* in the wild-type Col-0 background. (C) Arabidopsis PARG1 and PARG2 localized in the cytoplasm and nucleus. *35S*:*AtPARG1-GFP* and *35S*:*AtPARG2-GFP* were transiently expressed in *N*. *benthamiana* and the images were taken 2 d after inoculation.

To examine whether Arabidopsis PARPs are targeted to the nucleus as in animals, PARP1 and PARP2 proteins fusions to the C-terminus of green fluorescent protein (GFP) were expressed in leaves of *Nicotiana benthamiana* by agroinfiltration. Confocal fluorescence microscopy revealed that both PARP1 and PARP2 predominantly accumulate in the nucleus (Fig 4A). To determine the subcellular location of PARPs in Arabidopsis, we expressed PARP1-GFP and PARP2-GFP C-terminal fusion proteins in stable transformants of wild-type Col-0. Fluorescence microscopic examination of transgenic Arabidopsis plants expressing *35S*:*PARP1-GFP* or *35S*:*PARP2-GFP* detected PARP1 and PARP2 only in the nucleus, in both leaves and roots (Fig 4B), consistent with a previous report [50]. Notably, multiple foci were detected throughout the nucleus of root tissues expressing *35S*:*PARP2-GFP*.

Poly(ADP-ribosyl)ation is impacted by PARP enzymes and also by poly(ADP-ribose) glycohydrolase (PARG) enzymes that remove poly(ADP-ribosyl)ation, so experiments with PARG proteins were also carried out. PARG1-GFP and PARG2-GFP fusion proteins expressed in *N*. *benthamiana* were reproducibly observed both in the cytoplasm and nucleus (Fig 4C). PARG2 mRNA abundance was previously shown to be significantly increased in response to virulent or avirulent *Pseudomonas syringae* pv. *tomato* (*Pst*) strains or the PAMPs flg22 or elf18 [17, 41]. In the present study, up-regulation of PARG2 at the protein level was confirmed in transgenic Arabidopsis plants. The abundance of PARG2-GFP protein expressed under control of the *PARG2* promoter sequence was substantially increased in leaves within 8 hr after exposure to *Pst(avrRpt2)* (S7 Fig).

### *PARG1* (but not *PARG2*) mediates poly(ADP-ribose) removal in plants responding to bleomycin

**Fig 5:**
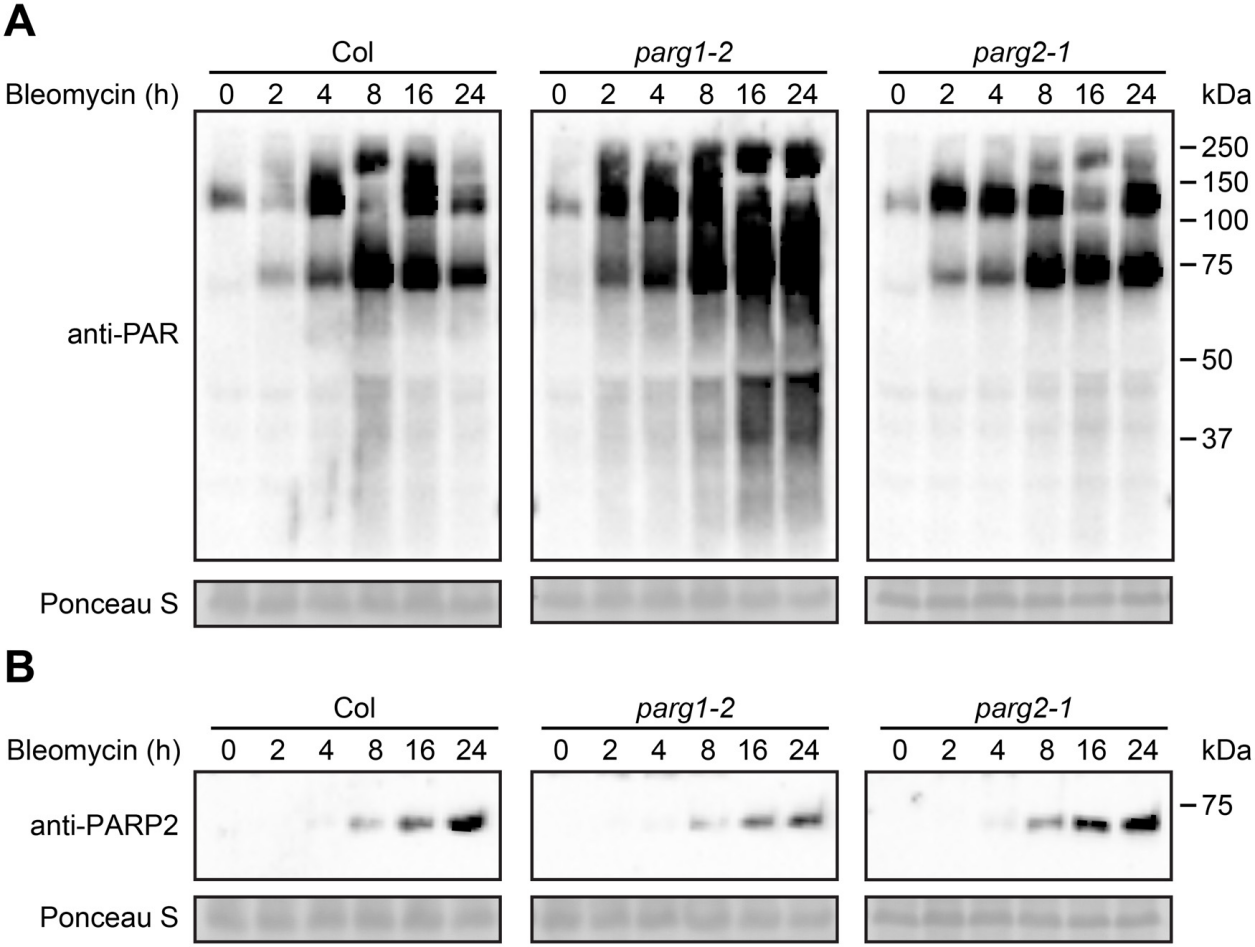
PARG1 is more active than PARG2 in removal of poly(ADP-ribosyl)ation after bleomycin treatment. Two-week-old *parg1-2*, *parg2-1* and Col-0 Arabidopsis plants were treated with 2.5 μg/ml bleomycin and samples were collected at indicated times. Total proteins were extracted, separated by SDS-PAGE and analyzed by immunoblotting with anti-PAR (A) or anti-PARP2 (B) antibody. Equivalent loading of lanes was verified using Ponceau S stain. Similar results obtained in two separate experiments.

Previous work by our group had detected multiple impacts on plant defense for Arabidopsis *parg1* mutants, unlike Arabidopsis *parg2* mutants [17, 41]. To determine if Arabidopsis *PARG1* and/or *PARG2* confer detectable poly(ADP-ribose) glycohydrolase activity, *parg1-1*, *parg2-1* and Col-0 plants were treated with 2.5 μg/ml bleomycin and the abundance of poly(ADP-ribosyl)ated proteins was examined. Mutation of *PARG1* resulted in significantly elevated presence of poly(ADP-ribosyl)ation in total protein extracts compared with wild-type plants, as might be expected for loss of an active poly(ADP-ribose) glycohydrolase (Fig 5A). Surprisingly but consistent with the Adams-Philips et al. data (2010), disruption of Arabidopsis *PARG2* caused little or no increase in poly(ADP-ribosyl)ation (Fig 5A). The data suggest that PARG1 is the primary enzyme that catalyzes the removal of poly(ADP-ribose) from acceptor proteins and that PARG2 makes little or no contribution to this activity. The primary mediator of bleomycin-induced elevation of poly(ADP-ribosyl)ation, PARP2, exhibited increased protein levels in response to bleomycin in all three genetic backgrounds. Notably, the accumulation of PARP2 induced by bleomycin is reduced in the *parg1-2* mutant relative to wild-type (Fig 5B), suggesting that PARG1 can influence the levels of PARP2 protein.

### PARP2 activity is regulated by PARP1 in response to DNA alkylating agent mitomycin C

**Fig 6:**
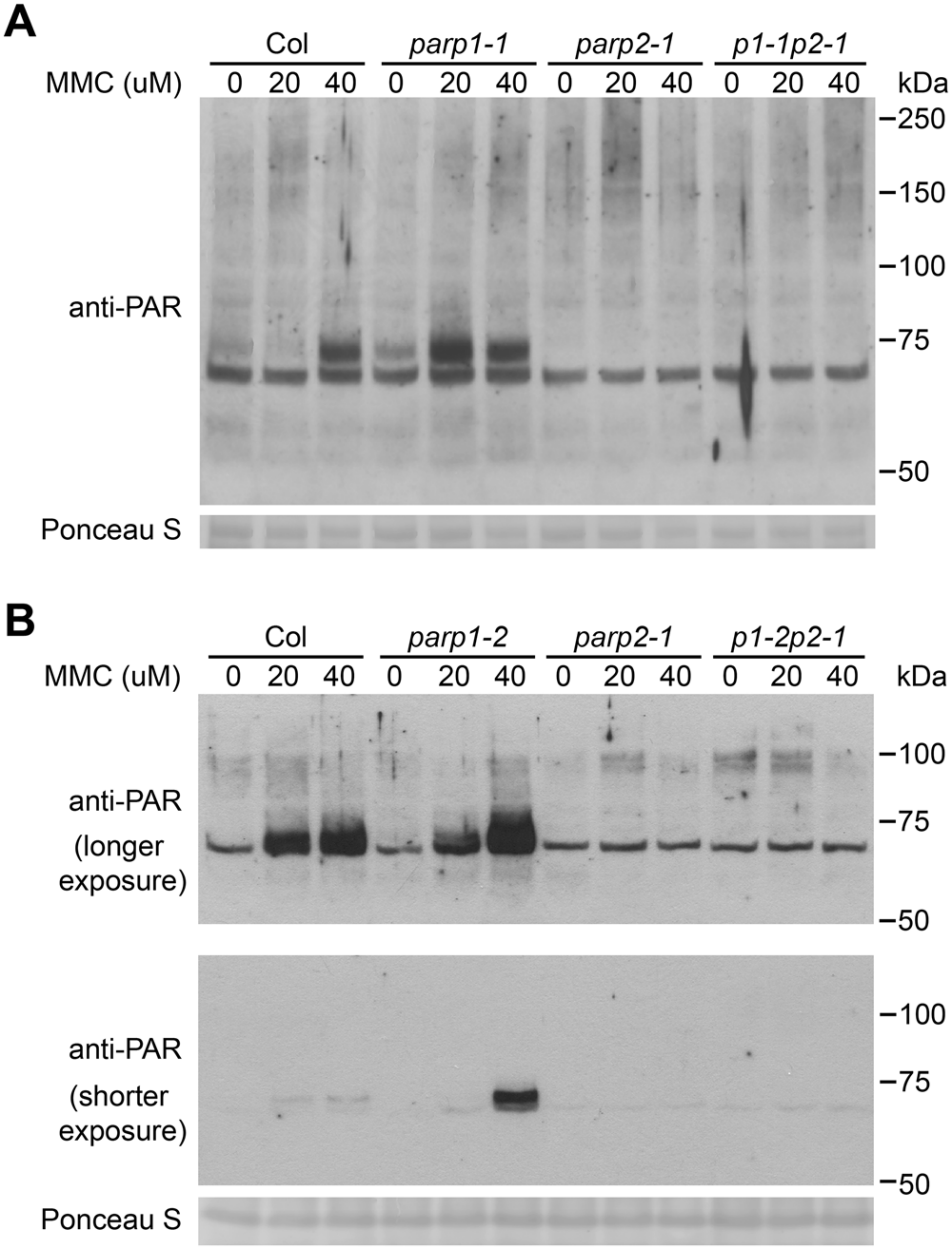
PARP2 activity is regulated by PARP1 in response to DNA alkylating agent mitomycin C. Arabidopsis plants, including (A) *parp1-1* or (B) *parp1-2* knockout alleles of *PARP1*, were grown on MS plates supplemented with 0, 20 and 40 μM of mitomycin C (MMC) for two weeks. Total proteins were extracted, separated by SDS-PAGE and analyzed by immunoblotting with anti-PAR antibody. Equivalent loading of lanes was verified using Ponceau S stain. Upper and middle panels of (B) are same blot, showing immunoblot signal after longer and shorter exposure times respectively. Similar results obtained in two separate experiments.

In further work to characterize the role of PARPs in plant DNA damage responses, wild-type and *parp* mutant plants were treated with mitomycin C to induce DNA cross-linking [51] and the level of poly(ADP-ribosyl)ated proteins was then monitored. As shown in Fig 6, mitomycin C caused increased PARP activity in wild-type plants. As with the bleomycin and γ-ray experiments (Figs 3 and 4), almost no poly(ADP-ribosyl)ation activity was detected after mitomycin C treatment in *parp2-1* mutant plants (Fig 6). However, increased rather than decreased abundance of poly(ADP-ribosyl)ation was observed in *parp1* mutants, in separate experiments with either the *parp1-1* or *parp1-2* alleles, in response to mitomycin C (Fig 6). This is unlike the poly(ADP-ribosyl)ation behavior of the same mutants in response to bleomycin or γ-irradiation (Fig 2). The mitomycin C-induced increase in poly(ADP-ribosyl)ation caused by mutation of PARP1 was eliminated if PARP2 was also mutated (*parp1-1parp2-1* and *parp1-2parp2-1* double mutants, Fig 6). This interesting finding suggests that loss of PARP1 can in some situations lead to elevated PARP2 activity. Said another way, PARP1 may suppress PARP2 activity under certain stress conditions such as after exposure to mitomycin C.

### PARP2 is required for normal basal resistance responses in Arabidopsis

**Fig 7:**
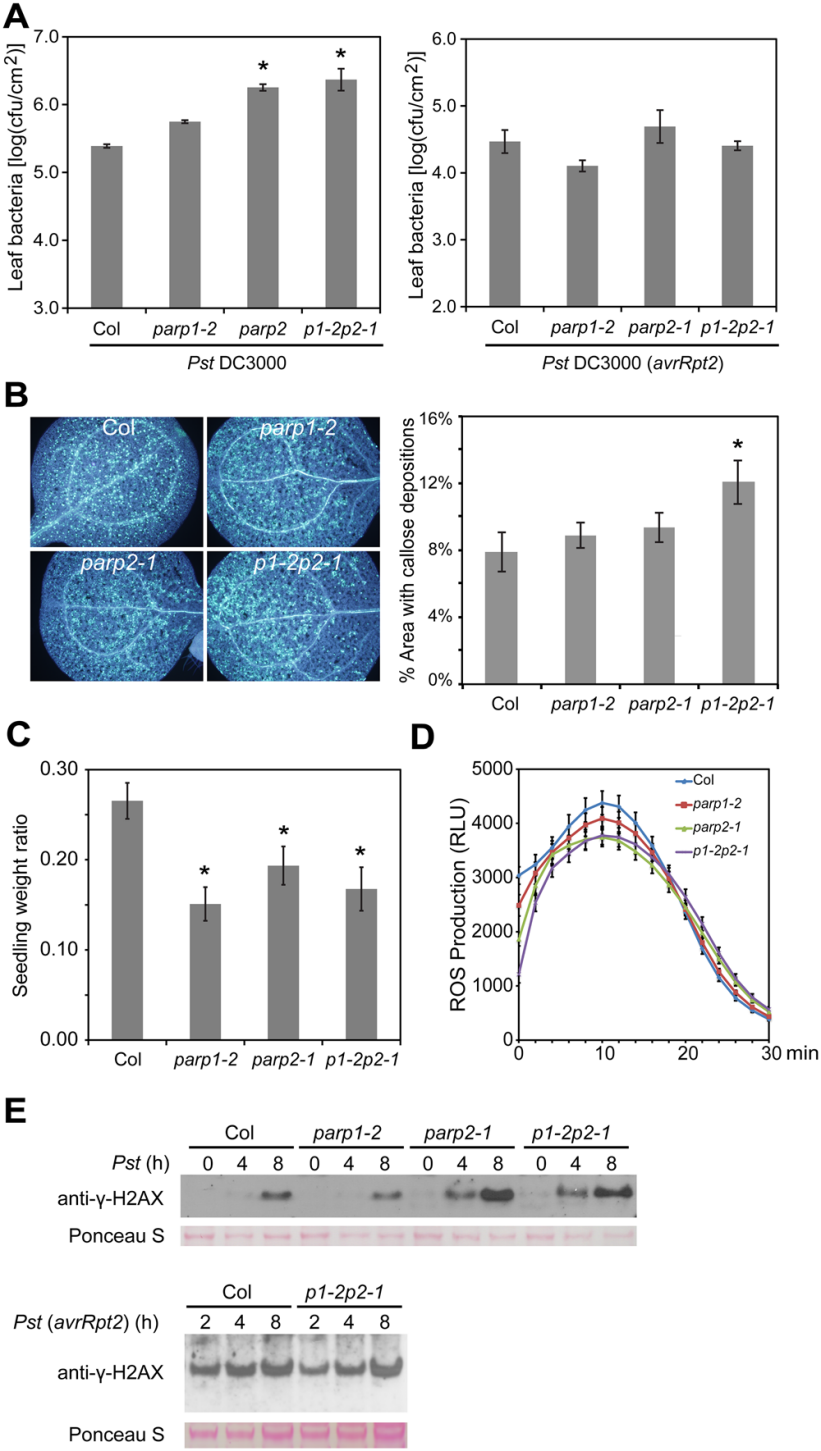
Arabidopsis *parp* mutants are compromised in basal resistance. (A) Bacterial population sizes of *Pst* within leaves. *Pst* DC3000 strains with or without *avrRpt2* were syringe infiltrated into leaf mesophyll at 1×10^5^ cfu/ml and bacterial populations were measured 3 d post-inoculation. Mean ± standard error of mean for one experiment shown. Experiments were performed three times with similar results; * indicates significant difference from Col-0 across the three experiments (ANOVA, Tukey pairwise comparisons, *P* < 0.05). (B) Flg22-induced callose deposition. Seedlings exposed to 1 μM flg22 for 24 h were fixed and stained with aniline blue to highlight callose deposition. Left panel: representative images of the four genotypes; right panel: data summary for all tested leaves (n = 24 per genotype). * indicates significant difference from Col-0 across the three experiments (ANOVA, Tukey pairwise comparisons, *P* < 0.05). (C) Seedling growth inhibition due to chronic flg22-induced defense activation. Ratio is weight of individual seedlings grown for 14 d in liquid MS media + 1 μM flg22, divided by mean of seedlings of same genotype grown without flg22 within same experiment (mean ± standard error of mean). * indicates significant difference from Col-0 across the three experiments (ANOVA, Tukey pairwise comparisons, *P* < 0.05). (D) Flg22-triggered oxidative burst. Reactive oxygen species from leaf discs of the indicated genotype were measured for 30 min after treatment with 1 μM flg22. RLU: relative luminescence units. (E) *Pst*-induced γ-H2AX accumulation. Arabidopsis plants of the indicated genotype were vacuum-infiltrated with the indicated *Pst* strain at 1×10^7^ cfu/ml. The level of γ-H2AX in leaf samples from the indicated time points after inoculation was assessed by immunoblot using anti-γ-H2AX antibody. Equivalent loading of lanes was verified using Ponceau S stain. Upper and lower blots are from separate experiments. Similar results obtained in two separate experiments.

To examine the role of *PARP* genes in plant defense responses, the Arabidopsis *parp* mutants were inoculated with the virulent bacterial pathogen *Pseudomonas syringae* pv. *tomato* (*Pst*) strain DC3000. As shown in Fig 7A, the *parp2-1* single and *parp1-2parp2-1* double mutants exhibited enhanced susceptibility in comparison to wild-type plants, whereas bacterial growth in the *parp1-2* single mutant was similar to that in wild-type. This important result demonstrates an impact of loss of poly(ADP-ribosyl)ation activity on the capacity of Arabidopsis to limit the growth of this virulent bacterial pathogen. The finding also suggests that wild-type PARP2 plays a greater role than PARP1 in basal defense against this pathogen.

Experiments with *Pst* DC3000 expressing the effector protein AvrRpt2 (which triggers RPS2-mediated defense in naturally *RPS2* ^*+*^ Arabidopsis Col-0 plants) were also conducted, to test for impacts of poly(ADP-ribosyl)ation on the stronger *R* gene-mediated defense response (also known as effector-triggered immunity) [52]. No impact of *PARP* mutations was detected (Fig 7A).

Impacts of *PARP* genes on PAMP-triggered immunity responses [52–54] were also examined. Callose deposition was not detectably affected in *parp1-2* or *parp2-1* single mutants, but callose deposition was significantly enhanced in the *parp1-2parp2-1* double mutant (Fig 7B). Enhanced callose deposition was also observed in *parp1-1parp2-1* double mutant plants (different *parp1* allele; S8 Fig). Reinforcing rather than antagonistic roles of PARP and PARG activity were suggested by the related observation that, like *parp1parp2* mutants, *parg1* mutant plants exhibited elevated callose deposition in response to flg22 (S9 Fig). Seedling growth inhibition assays [55], one of the most sensitive indicators of basal defense activation in response to PAMP treatment, also showed that disruption of PARP activity led to a stronger response to flg22 (Fig 7C). No significant difference in the flg22-induced ROS burst was observed for the *parp* mutants (Fig 7D).

We recently showed that microbial pathogens including virulent *Pst* DC3000 induce DNA DSBs in plant host genomes [1]. To monitor DSB induction in Arabidopsis *parp* mutants in response to *Pst*, we monitored the accumulation of γ-H2AX. Across replicate experiments, and consistent with the bacterial growth data of Fig 7A, no impact of *parp* mutations on DSB induction was observed during the strong *R* gene-mediated responses triggered by *Pst* DC3000(*avrRpt2*) (Fig 7E). However, with virulent *Pst* DC3000 lacking *avrRpt2*, elevated levels of γ-H2AX were observed 4 and 8 hours after infection in leaves of the *parp2-1* and *parp1-2parp2-1* mutants as compared to wild-type plants (Fig 7E). No increases of γ-H2AX were observed in *parp1-2* plants. These results indicate, as might be predicted [1], that there is a link between plant poly(ADP-ribosyl)ation and prevention or repair of pathogen-induced DNA damage. The results also indicate that in Arabidopsis, PARP2 plays a more significant role than PARP1 in this prevention/repair of pathogen-induced DNA damage.

### PARP1 interacts with PARP2; PARG1 interacts with PARG2; PARP1 and PARP2 interact with both PARG1 and PARG2

**Fig 8:**
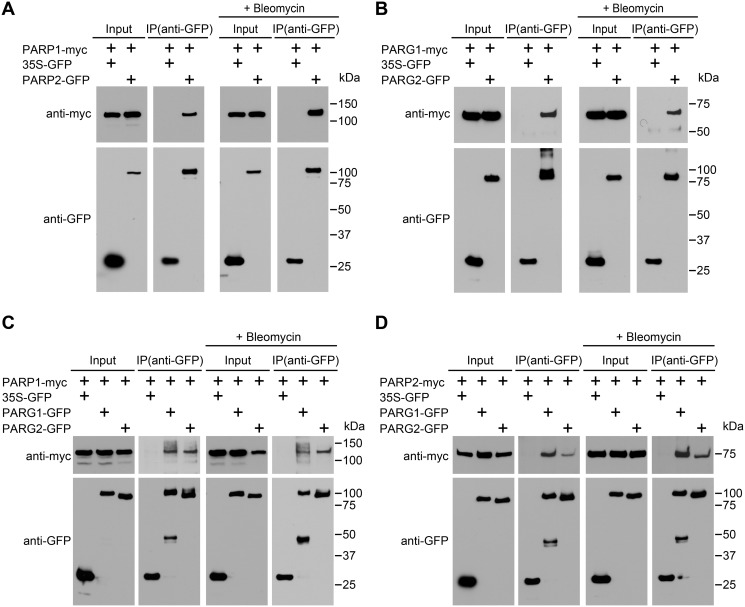
Interactions between PARPs and PARGs. (A) PARP1 associates *in vivo* with PARP2. (B) PARG1 associates *in vivo* with PARG2. (C) PARP1 associates *in vivo* with PARG1 and PARG2. (D) PARP2 associates *in vivo* with PARG1 and PARG2. The indicated proteins were transiently expressed in *N*. *benthamiana*. Input lanes were loaded with total protein extracts, IP lanes were loaded with immunoprecipitation products. Immunoprecipitations were performed with anti-GFP antibodies and immunoblots were analyzed with anti-GFP or anti-myc antibodies, as noted. These experiments were repeated twice with similar results.

To determine if plant PARP1 associates with PARP2, we carried out coimmunoprecipitation assays in *N*. *benthamiana*. As shown in Fig 8A, myc-tagged PARP1 was coimmunoprecipitated by GFP-tagged PARP2 using an anti-GFP antibody, indicating that at least some PARP1 is present in complexes with PARP2. The interaction was observed without as well as with bleomycin treatment. This readily detectable interaction suggests that PARP2 activity may be regulated in part by physical contact with PARP1. Similarly, PARG1 and PARG2 (the only two PARG proteins in Arabidopsis) also associated with each other (Fig 8B), although that interaction required longer exposures than the PARP1-PARP2 product of Fig 8A to detect a co-IP band of similar intensity. We also investigated whether PARPs interact with PARG1 and/or PARG2. The reproducibly detectable co-immunoprecipitation products in Fig 8C showed that PARP1 interacts with both PARG1 and PARG2, with or without bleomycin treatment. Similarly, PARP2 also complexes with both PARG1 and PARG2 (Fig 8D). Hence the regulation of PARP2 abundance and activity by PARG1 may also be achieved via physical interaction between the two proteins.

## Discussion

In mammals, PARP1 is by far the predominant contributor to poly(ADP-ribosyl)ation responses to a variety of cellular stresses, and mammalian PARP1 has received the vast majority of poly(ADP-ribose) polymerase research and pharmaceutical industry attention [7–9]. Arabidopsis PARP1 is the poly(ADP-ribose) polymerase with a similar domain structure to mammalian PARP1 (S1 Fig), but the relative roles of the different plant PARPs required investigation [11]. We used genetic and biochemical approaches to elucidate the contributions to Arabidopsis DNA damage and immune responses made by different poly(ADP-ribose) polymerase and poly(ADP-ribose) glycohydrolase gene products. For both groups of enzymes the predicted proteins have significantly divergent domain structures (S1 Fig). In contrast to the paradigm in mammals, we found that PARP2 rather than PARP1 plays the major role biochemically and in organismal-level responses to DNA damage and pathogen infections. Furthermore, we found that PARG1 rather than PARG2 is the primary enzyme that confers poly(ADP-ribose) glycohydrolase activity in Arabidopsis during the tested responses to DNA damage and pathogen infection.

Arabidopsis PARP1 shares a substantially conserved domain structure with human PARP1, whereas Arabidopsis PARP2 is more analogous to human PARP2. In mice, *parp2* mutants exhibited some phenotypes similar to *parp1* knockouts, despite the dramatic difference between their respective specific enzymatic activities [56–58]. Although neither PARP1 nor PARP2 is required for viability in mice, *parp1parp2* double knockouts are embryonic lethal with considerable genomic instability, indicating that the PARP1 and PARP2 gene products together are essential during early embryogenesis and that the deficiency in PARP1 and PARP2 cannot be functionally compensated by other PARP family members [59]. In contrast, when the functions of Arabidopsis PARP1 and PARP2 both were disrupted plants were developmentally normal, demonstrating that this pair of genes is not essential to viability in Arabidopsis

Overall PARP activity in Arabidopsis is significantly decreased when PARP2 is knocked out, suggesting that Arabidopsis PARP2, unlike its counterpart in animals, is responsible for majority of the PARP activity. The predominant role of Arabidopsis PARP2 was detected at more macroscopic levels by the increased sensitivity of *parp2* mutant plants to genotoxic stresses, and enhanced susceptibility to virulent *Pst* growth and to *Pst*-induced DNA DSB damage. Since poly(ADP-ribosyl)ated proteins were nearly absent in the *parp1parp2* double mutant, it is reasonable to propose that other Arabidopsis PARP-domain containing proteins have either low or no PARP activity under the conditions examined in this study. This is consistent with the bioinformatic prediction that Arabidopsis PARP3, RCD1 and SROs lack conserved active catalytic sites in the PARP domains and possibly have lost the ability to bind NAD [12,19]. We had previously reported that *P*. *syringae* induces the accumulation of poly(ADP-ribosyl)ated proteins [17] but we know of no studies that have identified the suite of proteins that are poly(ADP-ribosyl)ated during plant-pathogen interactions.

PARP1, the primary PARP that mediates responses such as DNA damage repair in human cells, contains zinc-finger DNA binding domains that are crucial to its function [10, 24] (S1 Fig). Known DNA binding domains are absent from human PARP2. The striking finding that core poly(ADP-ribosyl)ation functions of animal PARP1 have been taken on by PARP2 in plants may be less unusual in light of the fact that plant PARP2 proteins carry N-terminal SAP (SAF-A/B, Acinus, and PIAS) domains (S2 Fig). The SAP domain is a highly conserved sequence-specific or structure-specific DNA binding motif with a four-helix bundle, known to contribute to regulation of chromatin structure and transcription [60, 61]. In contrast to many other DNA recognition protein structures, one end of the helix bundle makes contact with DNA and fits into the minor groove of DNA [62]. One to four distinct SAP domains are present in plant PARP2s, depending on the plant species (S2 Fig). Structural differences in DNA binding domains between Arabidopsis PARP1 and PARP2 likely contribute intriguing differences in the substrate specificities of these two enzymes. Arabidopsis PARP1 and PARP2 also exhibit substantial divergence within their areas of shared domain structure (see Fig 1; for amino acids #130–636 of Arabidopsis PARP2, only 56% are similar (36% identical) to the aligned Arabidopsis PARP1 amino acids #485–982). The substantially divergent structure of plant PARP2 enzymes is likely to be accompanied by substantially divergent mechanisms of regulation.

We found evidence that Arabidopsis PARP2 activity is negatively regulated by PARP1 during responses to mitomycin C, but we did not detect reproducible changes of PARP2 protein abundance in the *parp1* mutant under the genotoxic stress conditions we examined. However, it has recently been reported that Arabidopsis PARP1 and PARP2 negatively regulate gene expression of each other [34]. We observed physical interaction (coimmunoprecipitation) of PARP1 and PARP2 in plants responding to bleomycin. The Boltz et al. data and our data point to an intriguing interplay between PARP1 and PARP2, and also suggest that different modes of action of poly(ADP-ribosyl)ation-mediated regulation may exist in plants that are not observed in animals.

In animals, poly(ADP-ribosyl)ation has recently been found to play a significant role during host-pathogen interactions. For example, *Helicobactor pylori*, a human gastric bacterial pathogen, activates nuclear regulator PARP1 [63]. Several proteins produced by pathogenic viruses have been reported to interact directly with and stimulate animal PARP1 enzymatic activity [64, 65], whereas others prevent PARP1 activation as virulence strategies [66, 67]. In plants, there is also an expanding body of evidence that poly(ADP-ribosyl)ation plays critical roles in pathogenicity as well as in immune responses [17, 41, 68–70]. The present study found that PARP1/2 are positive regulators in plant immune response. The molecular mechanisms by which PARPs regulate plant immunity remain to be fully discovered, but in light of the γ-H2AX findings summarized in Fig 7E, it would appear that one important role of Arabidopsis PARP2 is to minimize the host DNA damage elicited by virulent pathogens such as *Pst*.

Over the past 30 years, the function of PARPs in maintenance of genome integrity have been extensively characterized [7, 8]. In contrast, study of PARG in poly(ADP-ribosyl)ation has been limited, due in part to its low cellular abundance and high sensitivity to proteases [71], but also to the lethality of knockouts of the sole *PARG* gene in metazoans [37, 38]. The presence of two *PARG* genes in plants may offer opportunities for genetic and molecular investigation not available in animal systems. Using Arabidopsis *parg* null mutants, which are viable, we demonstrated that PARG1 mediates removal of poly(ADP-ribose) whereas PARG2 confers limited PARG activity in the examined conditions. This is in line with the observation that *parg1* plants, but not *parg2* plants, exhibit increased sensitivity to PAMP treatment and grow less well than wild-type plants in response to DNA-damaging mitomycin C [17]. Because multiple but divergent copies of *PARG* genes are found in a number of plant species [11], in the future it will be interesting to explore if similar divisions of labor among PARG proteins are common in other species, and if PARG proteins from other plant species differ substantially from those of Arabidopsis in their enzymatic activities and cellular roles.

PARPs and PARGs, enzymes with counteracting enzymatic activities, modify target proteins by addition or removal, respectively, of ADP-ribose polymers. Surprisingly, the present and previous studies showed that *parg* and *parp* mutant plants exhibit some unexpectedly similar phenotypes, such as strong seedling growth inhibition and increased callose deposition [17, 41]. This may be attributable to synergistic functions of PARPs and PARGs. There is evidence that in human cells the two enzymes co-localize to target gene promoters and act with a similar rather than antagonistic overall effect to regulate gene expression globally [72]. PARP1 and PARG also function in concert to accelerate single-strand break repair in human cells [73]. Intriguingly, we discovered that the accumulation of PARP2 protein in response to genotoxic agents was reduced relative to wild-type in loss-of-function *parg1* mutant plants, consistent with a recent finding made in human HeLa cells that PARP1 transcript and protein expression levels decreased in the *parg* knockdown. This suggests that PARP1 is regulated by PARG to avoid excess accumulation of poly(ADP-ribose) in a cell [74]. With our detection of plant PARPs and PARGs in the same *in vivo* complexes, it is plausible to hypothesize that regulation of abundance or activity is achieved in part through physical interactions between PARPs and PARGs [16, 75].

In summary, we have found that although plant PARPs and PARGs have partially overlapping functions Arabidopsis PARP2 and PARG1 play the predominant roles in plant poly(ADP-ribosyl)ation during DNA damage and immune responses. Future studies will further identify the molecular mechanism by which PARPs and PARGs regulate various cellular responses, individually or in a concerted manner, and their functional interplay with each other. Identification of the proteins that are poly(ADP-ribosy)ated is another future research direction that may help to elucidate the regulatory functions of plant poly(ADP-ribosyl)ation, both under normal physiological conditions and in response to the stresses of DNA damage or pathogenic infection.

## Materials and Methods

### Plant materials and growth conditions

*Arabidopsis thaliana* plants were grown in Fisons Sunshine Mix #1 soil-less potting mix (Hummert) at 22°C under 9-h light/15-h dark cycles, or MS-grown plants were cultivated on Murashige-Skoog (MS) agar media at 22°C under 16-h light/8-h dark cycles.

The homozygous Arabidopsis T-DNA insertion lines *parp1-1* (GABI_380E06), *parp1-2* (GABI_382F01), *parp2-1* (GABI_420G03), all in the Col-0 background [76], were identified as previously described [77]. Isolation and initial characterization of the *parg1* and *parg2* T-DNA insertion lines was previously described [17]. Homozygous double-mutant lines were obtained as self-fertilized progeny from crosses of single mutants and were identified using PCR-based allele-specific markers.

### Mitomycin C and bleomycin sensitivity assay

Seeds were stratified at 4°C for two d and then approximately 100 seeds were grown for 14 d on each MS agar plate containing different concentrations of mitomycin C (0, 15 and 30 μM) and bleomycin (0, 1, 1.5 μg/ml) (both from Sigma-Aldrich), then the number of plants with and without true leaves was recorded. Sensitivity was scored as the percentage of plants without true leaves, using three or four plates for each concentration (or in a few instances two, due to plate contamination) within each experiment, and the entire experiment was repeated three separate times. Mean and standard error for each chemical concentration are reported from within each experiment.

### Seedling growth inhibition assay

To monitor plant defense activation in response to the flagellin epitope flg22 peptide, 6 d old seedlings from MS plates were transferred to 1 ml MS liquid medium + 1 μM flg22 in 24-well plates, and seedling fresh weights were recorded 14 d later for 12 seedlings per treatment, as per [55].

### ROS assay

The burst of reactive oxygen species produced in response to the flagellin epitope flg22 peptide was monitored as previously described [78]. Briefly, leaf discs were taken from 5-week-old plants and incubated in 1% dimethyl sulfoxide (DMSO) solution in a 96-well plate overnight, then treated with 1 μM flg22 in 0.1 mg/ml luminol and 0.1 mg/ml horseradish peroxidase immediately prior to 30 min. of luminescence measurement by plate reader (Centro XS^3^ LB 960, Berthold Technology).

### Callose deposition

Arabidopsis seedlings were grown on MS agar plates for five days before being transferred to liquid MS containing 1 μM flg22. After 24 h of treatment, seedlings were fixed in FAA solution (10% formaldehyde, 5% acetic acid and 50% ethanol), cleared in 95% ethanol, and stained with 0.01% aniline blue in 67 mM K_2_HPO_4_ with pH adjusted to 12. The stained seedlings were visualized with an Olympus BX60 Epifluorescence Microscope and images of entire cotyledons were captured with an Olympus DP73 camera (with same settings used throughout single experiment). The callose deposits on entire cotyledons were then quantified automatically using ImageJ software by excluding rare wounded leaves and then analyzing the entire area of all images within an experiment after setting ImageJ hue and brightness cutoff levels using images for positive and negative control leaves.

### Bacterial growth assay

Leaves of healthy five-week-old Arabidopsis plants were syringe-inoculated with *Pst* DC3000 or *Pst* DC3000(*avrRpt2*) at 1×10^5^ cfu/ml [79]. After 3 d, leaf discs were sampled from inoculated plants and macerated in 10 mM MgCl_2_. Samples were diluted serially, plated on NYGA plates with rifampicin and kanamycin and the number of colonies was recorded after 2 d incubation at 28°C.

### Ionizing radiation and detection of H2AX phosphorylation

For ionizing radiation, Arabidopsis Col-0 plants were irradiated with 150 Gy from a ^137^Cs source (administered at 2.14 Gy per minute) and tissue samples were collected at the indicated times after removal from the radiation source [47]. To detect pathogen-induced DSBs, 5-week-old plants were infiltrated with a 1×10^7^ cfu/ml solution of *Pst* bacteria in 10 mM MgCl_2_ and samples were collected at indicated times. Histone proteins were prepared from leaf tissues as previously described and were subjected to immunoblotting with rabbit anti-human γ-H2AX antibody at 1:5000 dilution (Sigma-Aldrich).

### Confocal laser scanning microscopy

The full-length cDNAs of PARP1/2 and PARG1/2 were subcloned into the pDONR 207 vector (Invitrogen) and introduced into the destination vector pGWB405, resulting in constructs with C-terminal fusions to GFP under the control of 35S promoter. The sequence-verified constructs were transformed into *Agrobacterium tumefaciens* strain GV3101(pMP90) and transiently expressed in *Nicotiana benthamiana* leaves by infiltration. Confocal fluorescence microscopy was carried out at indicated times using a Zeiss 510 Meta confocal laser scanning microscope. *Agrobacterium* strains carrying *35S*:*PARP1-GFP* and *35S*:*PARP2-GFP* were also used to transform wild-type Col-0 Arabidopsis by floral dip [80]. Stable transgenic T2 lines were selected and the subcellular locations of PARP1 and PARP2 were examined by confocal microscopy.

### Complementation of the *parp2* mutants

To complement the *parp2* mutants, *PARP2* genomic DNA fragments including its native promoter was cloned into the pDONR207 (Invitrogen). Using LR reactions, PARP2 was cloned into pGWB3300, a modified vector (Y. Cao and A. Bent) carrying in the multiple cloning site of pCambia3300 (http://www.cambia.org) the Gateway cloning site, C-terminal 3xHA and *nos* terminator sequences from pGWB13 [82]. This resulted in the generation of *PARP2*:*PARP2-3×HA* construct that was then transformed into the *parp2-1* mutant background.

### Immunoblot analysis

Total proteins were prepared from Arabidopsis plants in extraction buffer (50 mM Tris-HCl (pH7.5), 150 mM NaCl, 5 mM EDTA, 0.5% Triton X-100, 10% glycerol, and Sigma-Aldrich plant protease inhibitor cocktail at 1:100). After protein separation by SDS-PAGE, immunoblot analysis was carried out with anti-HA (Roche), anti-poly(ADP-ribose) (anti-PAR) (Trevigen) or anti-PARP2 antibodies. Polyclonal antibodies to Arabidopsis PARP2 protein (custom purchase from Genscript) were raised in rabbit against the synthetic peptide YGKEENDSPVNNDI, which does not share significant homology with PARP1 or other proteins predicted in the Col-0 accession of Arabidopsis. Anti-PARP2 antibodies were purified by peptide affinity chromatography.

### Coimmunoprecipitation assays

The cDNAs of PARP1, PARP2, PARG1 and PARG2 were cloned into the GFP-tagged pGWB405 and/or myc-tagged pGWB417 Gateway destination vectors [81] and the resulting constructs were transformed into *A*. *tumefaciens* GV3101(pMP90). Leaves of 4–5 week-old *N*. *benthamiana* plants were agroinfiltrated with OD_600_ 0.4 of the resulting *A*. *tumefaciens* strains, and some samples were then infiltrated two days later with 2 μg/ml of bleomycin solution. Tissues were harvested three days after agroinfiltration and total proteins were prepared in extraction buffer (50 mM Tris-HCl (pH7.5), 150 mM NaCl, 5 mM EDTA, 0.2% Triton X-100, 10% glycerol, and Sigma-Aldrich plant protease inhibitor cocktail at 1:100). Immunoprecipitation was carried out with anti-GFP (Abcam) at 4°C overnight followed by incubation with protein A beads (Thermo Scientific) for 1–2 h. The beads were washed three times with extraction buffer without protease inhibitors. The precipitated proteins were eluded with the SDS loading buffer, subjected to SDS-PAGE and immunoblotted with anti-myc (Covance) and anti-GFP (Clontech) antibodies, and detected using Supersignal West Pico or Dura chemiluminescent substrates (Thermo Scientific).

## Supporting Information

S1 Fig Domain structures of PARPs and PARGs. (A). Domain structures of human and Arabidopsis poly(ADP-ribose) polymerases. Zn1, Zn2 and Zn3: three zinc binding domains; BRCT: BRCA-1 C-terminal domain for phospho-protein binding. WGR: conserved Trp-Gly-Arg motif for putative nucleic acid binding; PRD: PARP regulatory domain; PARP: PARP catalytic domain; SAP: SAF-A/B, Acinus and PIAS motif for putative DNA/RNA binding. (B) Domain structures of human and Arabidopsis poly(ADP-ribose) glycohydrolases. A-domain: N-terminal regulatory and targeting domain; MTS: mitochondrial targeting sequence; Catalytic domain: PARG catalytic domain. Protein structures were generated using DOG 2.0 software.
(TIF)

S2 Fig Domain organization of plant PARP2 family. Domain structures of plant PARP2 proteins were identified by Phytozome (http://www.phytozome.net) using Arabidopsis PARP2 as a query. Color codes for domains are: Green for SAF-A/B, Acinus and PIAS (SAP) motif putative DNA/RNA binding domain; Blue for Trp-Gly-Arg (WGR in single letter code) putative PARP nucleic acid binding domain; Orange for PARP regulatory domain; Red for PARP catalytic domain. Number of amino acids in PARP proteins and names of plant species from which they derive are also shown.
(TIF)

S3 Fig Characterization of PARP T-DNA insertion lines. RT-PCR analysis of *PARP1* and *PARP2* mRNA in 3-week old wild-type Arabidopsis Col-0, or in *parp1* or *parp2* mutants. *Actin-2* amplified from the same RNA samples served as an RNA isolation and RT-PCR control.
(TIF)

S4 Fig Arabidopsis *parp* mutants are hypersensitive to DNA damage agents. Wild-type Col-0 and *parp* mutant (including *parp1-1* allele) seeds were grown on MS agar medium supplemented with the genotoxic agent mitomycin C. Sensitivity to DNA damage agents was scored as the percentage of plants that had not yet developed true leaves after 14 d. Mean and standard error of the mean are shown for one experiment; experiment was performed three times with similar results.
(TIF)

S5 Fig Analysis of PARP2 proteins in T-DNA insertion lines. PARP2 protein in 2-week old Arabidopsis seedlings of indicated genotypes, left untreated or treated with 5 μg/ml bleomycin for 18 h. Total proteins were extracted, separated by SDS-PAGE and detected with anti-PARP2 antibody. * Equivalent loading of total protein was verified using the signal from a high MW protein recognized by the polyclonal antibody.
(TIF)

S6 Fig Complementation of the *parp2-1* mutation with *PARP2*:*PARP2-HA*. *PARP2*:*PARP2-HA* was transformed into the *parp2-1* background. Five independent T2 lines, as well as *parp2-1* and wild-type Col-0 plants included as negative and positive controls, were treated with 2.5 μg/ml of bleomycin for 18 h. Total proteins were extracted, separated by SDS-PAGE and detected with anti-poly(ADP-ribose) or anti-PARP2 antibody as indicated. Equivalent loading of lanes was verified using Ponceau S stain.
(TIF)

S7 Fig PARG2 protein is upregulated by *Pst* DC3000(*avrRpt2*). Five-week-old Arabidopsis *parg2-1* mutant plants carrying *PARG2*:*PARG2-GFP* (2 kb of *PARG2* promoter, *PARG2* coding sequence fused to C-terminal GFP and *nos* terminator) were infiltrated with 10 mM MgCl_2_, or *Pst* DC3000(*avrRpt2*) at a concentration of 1×10^7^ cfu/ml in 10 mM MgCl_2_. Proteins were extracted at the indicated times, separated by SDS-PAGE and detected with anti-GFP antibody. Equivalent loading of lanes was verified using Ponceau S stain. Hypersensitive response-associated cell death is present in Arabidopsis Col-0 leaf tissues 24 h after infection by *Pst avrRpt2*.
(TIF)

S8 Fig Callose deposition phenotypes of *parp* mutants. Seedlings exposed to 1 μM flg22 for 24 h were fixed and callose deposits were detected using aniline blue staining and quantified by ImageJ software. * indicates significant difference from Col-0 across the three experiments (ANOVA, Tukey pairwise comparisons, *P* < 0.05).
(TIF)

S9 Fig Callose deposition phenotypes of *parg* mutant. Seedlings exposed to 1 μM flg22 for 24 h were fixed and callose deposits were detected using aniline blue staining and quantified by ImageJ software. * indicates significant difference from Col-0 across the three experiments (ANOVA, Tukey pairwise comparisons, *P* < 0.05).
(TIF)
