# Supplementary figures and images for: PARP2 Is the Predominant Poly(ADP-Ribose) Polymerase in Arabidopsis DNA Damage and Immune Responses
Source: PLoS Genet. 2015 May 7;11(5):e1005200. doi: 10.1371/journal.pgen.1005200 (PMC4423837; doi:10.1371/journal.pgen.1005200)

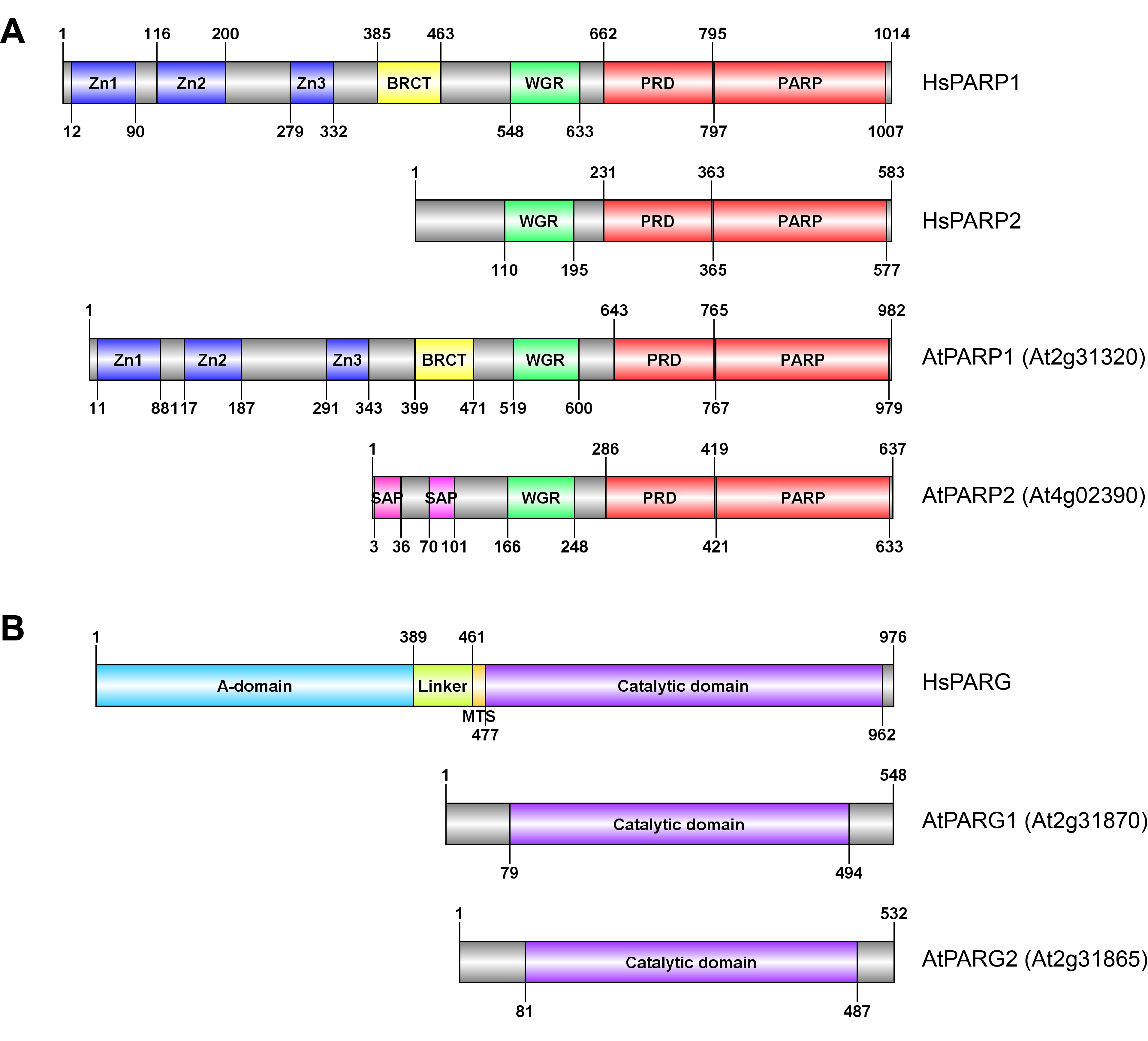

Supplement: S1 Fig — (A). Domain structures of human and Arabidopsis poly(ADP-ribose) polymerases. Zn1, Zn2 and Zn3: three zinc binding domains; BRCT: BRCA-1 C-terminal domain for phospho-protein binding. WGR: conserved Trp-Gly-Arg motif for putative nucleic acid binding; PRD: PARP regulatory domain; PARP: PARP catalytic domain; SAP: SAF-A/B, Acinus and PIAS motif for putative DNA/RNA binding. (B) Domain structures of human and Arabidopsis poly(ADP-ribose) glycohydrolases. A-domain: N-terminal regulatory and targeting domain; MTS: mitochondrial targeting sequence; Catalytic domain: PARG catalytic domain. Protein structures were generated using DOG 2.0 software. (TIF) [file pgen.1005200.s001.tif]

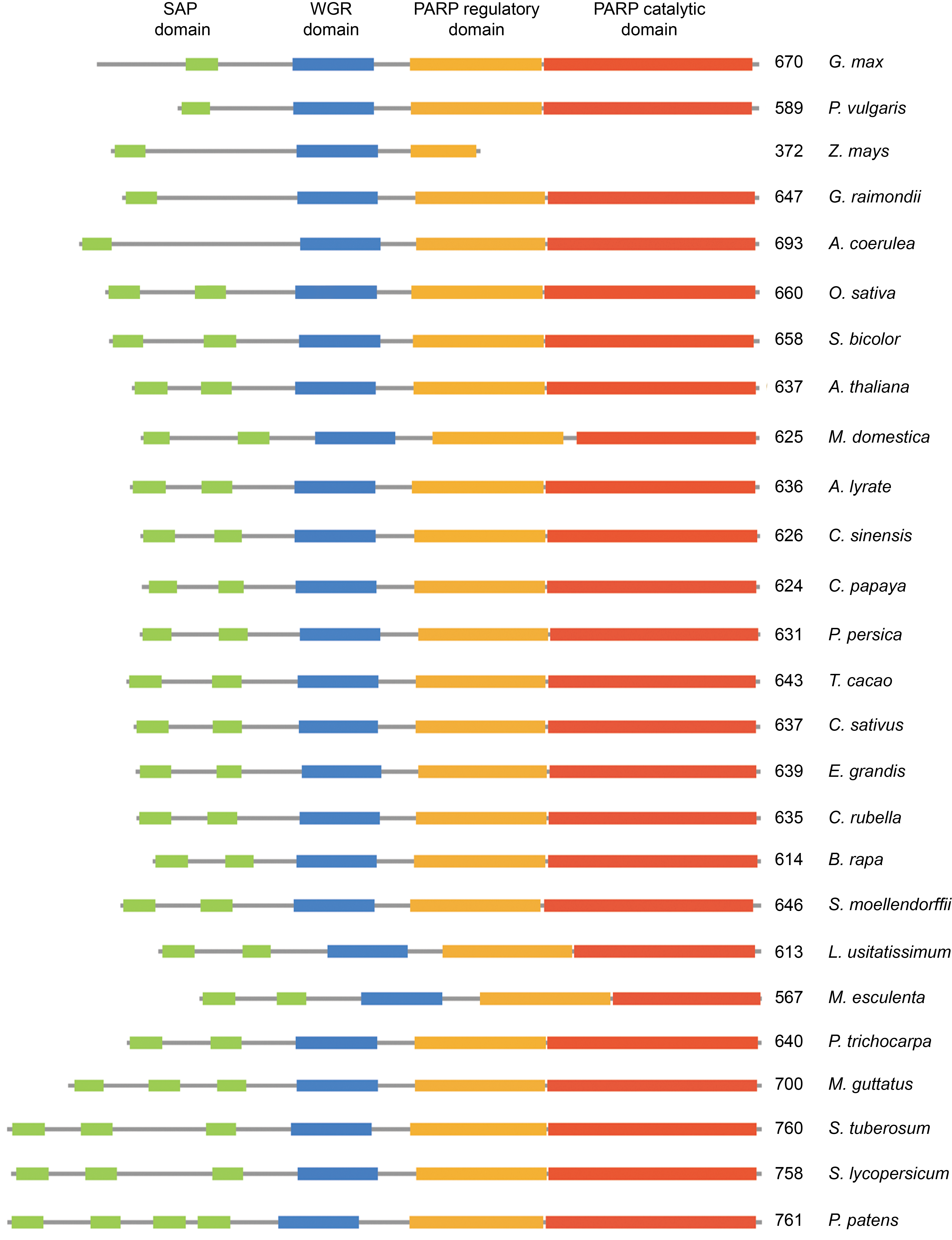

Supplement: S2 Fig — Domain structures of plant PARP2 proteins were identified by Phytozome (http://www.phytozome.net) using Arabidopsis PARP2 as a query. Color codes for domains are: Green for SAF-A/B, Acinus and PIAS (SAP) motif putative DNA/RNA binding domain; Blue for Trp-Gly-Arg (WGR in single letter code) putative PARP nucleic acid binding domain; Orange for PARP regulatory domain; Red for PARP catalytic domain. Number of amino acids in PARP proteins and names of plant species from which they derive are also shown. (TIF) [file pgen.1005200.s002.tif]

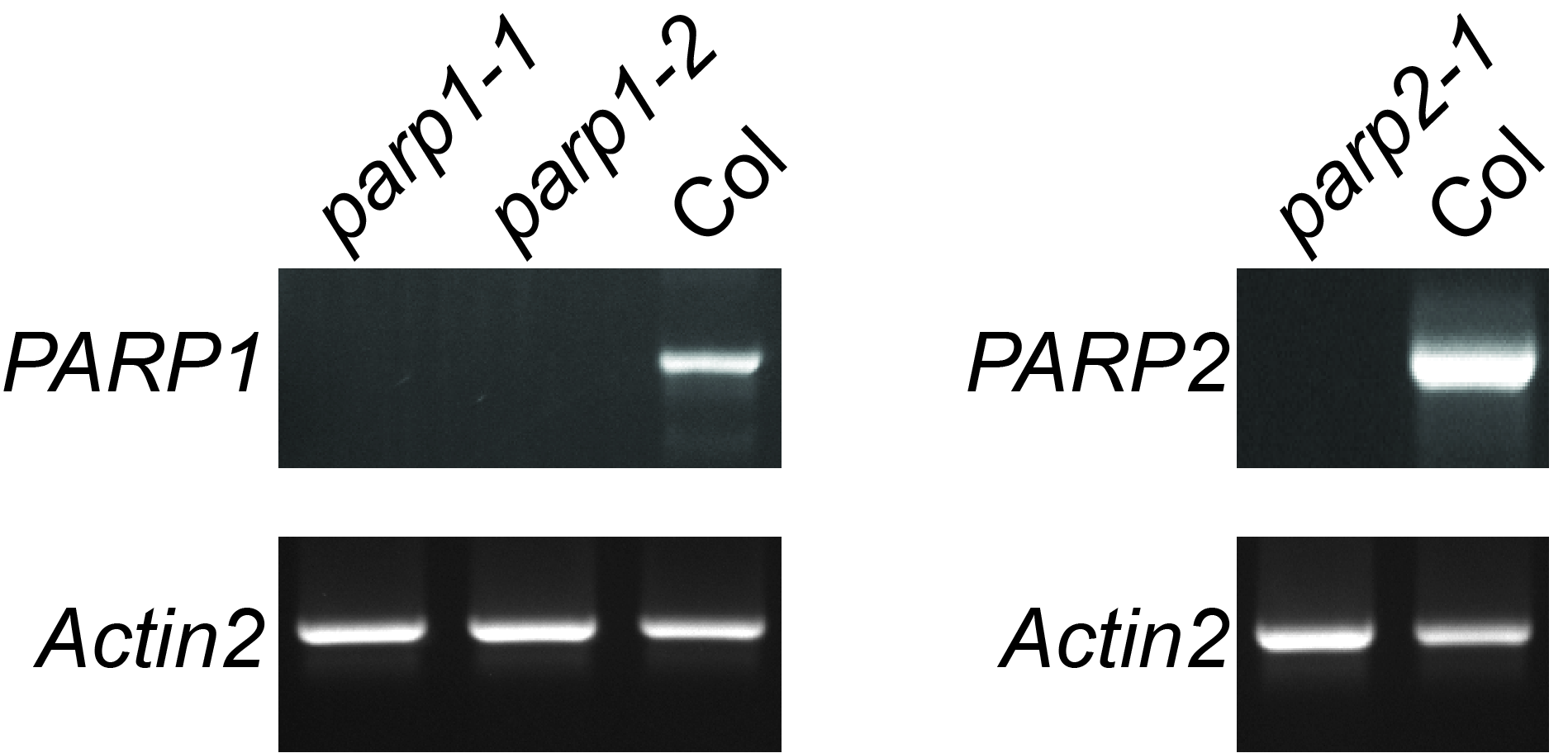

Supplement: S3 Fig — RT-PCR analysis of PARP1 and PARP2 mRNA in 3-week old wild-type Arabidopsis Col-0, or in parp1 or parp2 mutants. Actin-2 amplified from the same RNA samples served as an RNA isolation and RT-PCR control. (TIF) [file pgen.1005200.s003.tif]

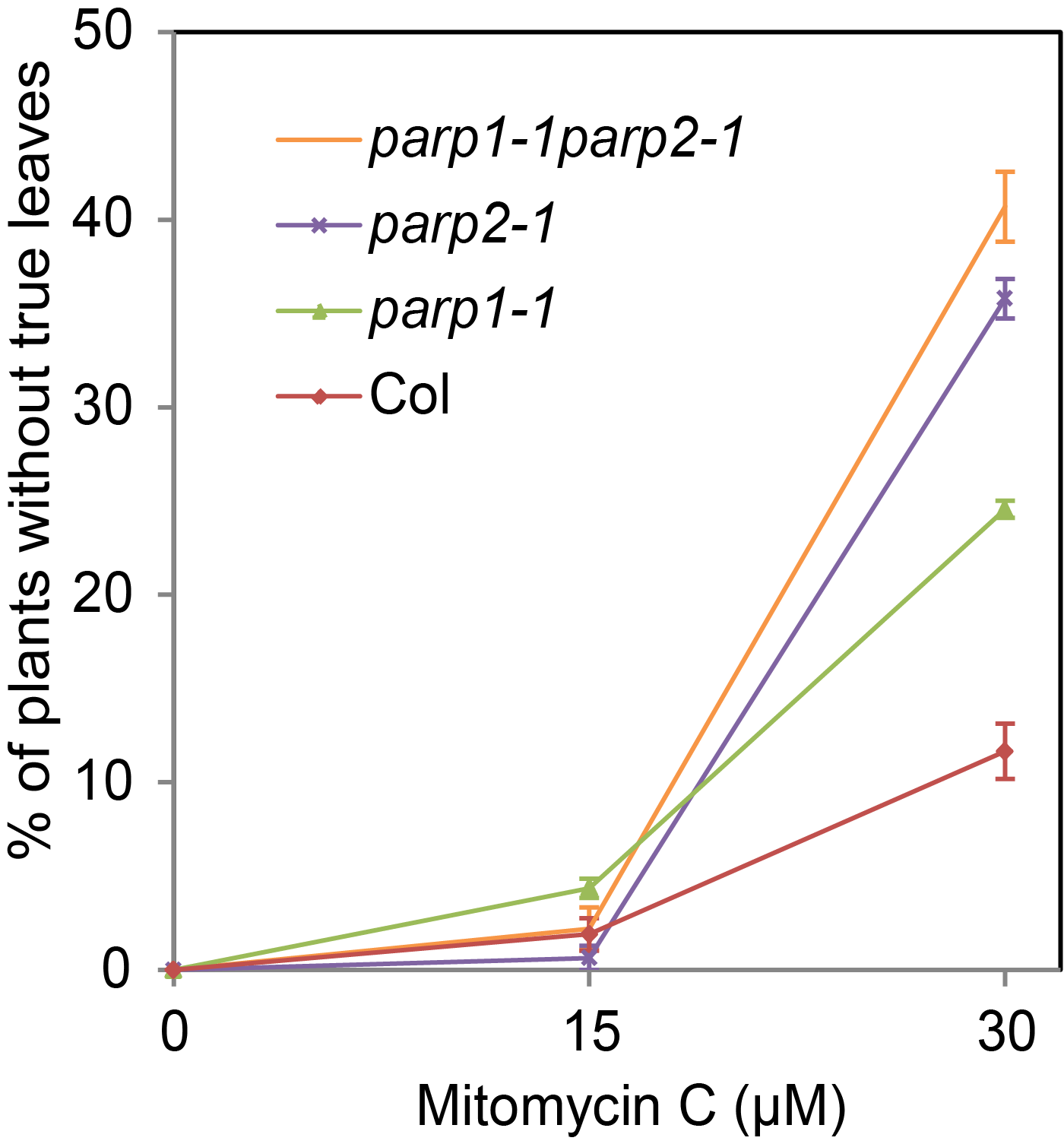

Supplement: S4 Fig — Wild-type Col-0 and parp mutant (including parp1-1 allele) seeds were grown on MS agar medium supplemented with the genotoxic agent mitomycin C. Sensitivity to DNA damage agents was scored as the percentage of plants that had not yet developed true leaves after 14 d. Mean and standard error of the mean are shown for one experiment; experiment was performed three times with similar results. (TIF) [file pgen.1005200.s004.tif]

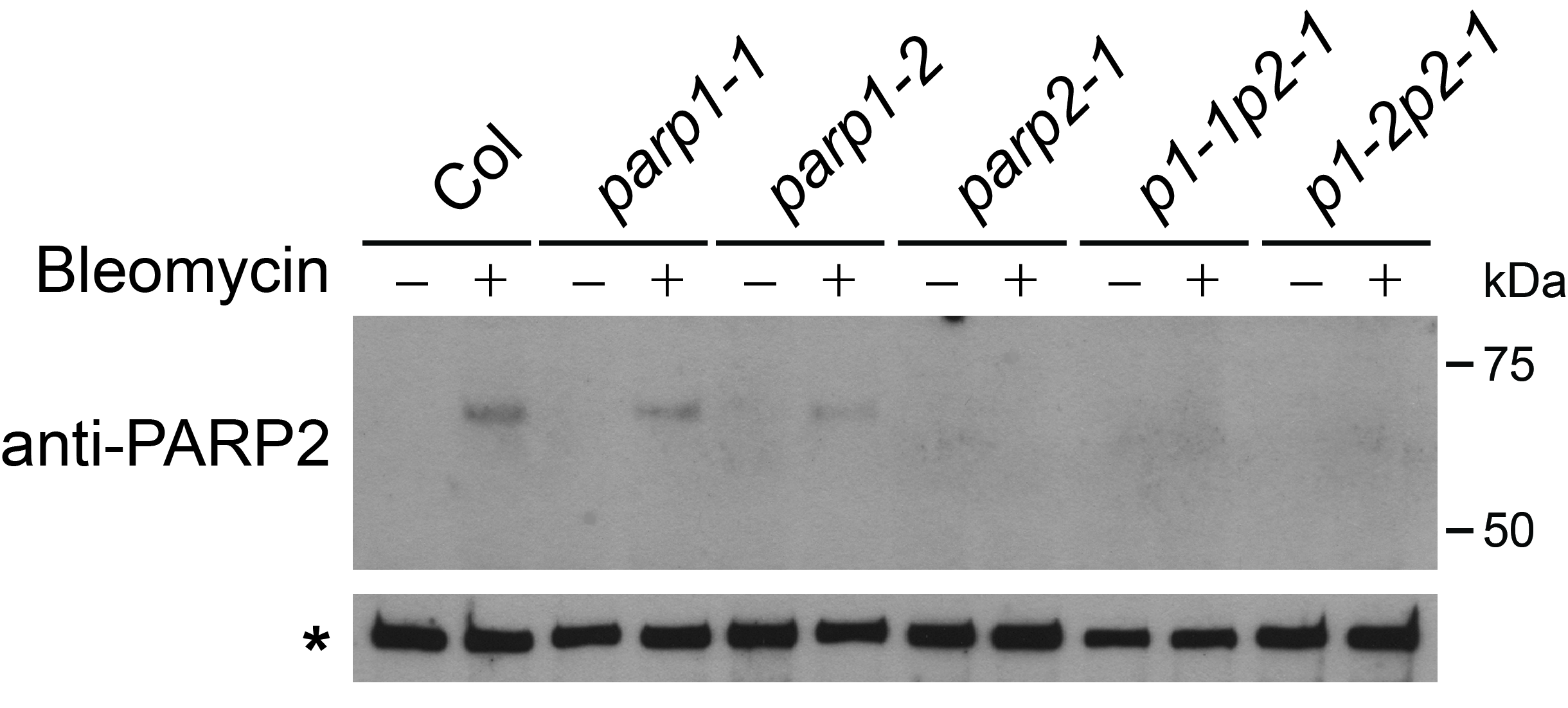

Supplement: S5 Fig — PARP2 protein in 2-week old Arabidopsis seedlings of indicated genotypes, left untreated or treated with 5 μg/ml bleomycin for 18 h. Total proteins were extracted, separated by SDS-PAGE and detected with anti-PARP2 antibody. * Equivalent loading of total protein was verified using the signal from a high MW protein recognized by the polyclonal antibody. (TIF) [file pgen.1005200.s005.tif]

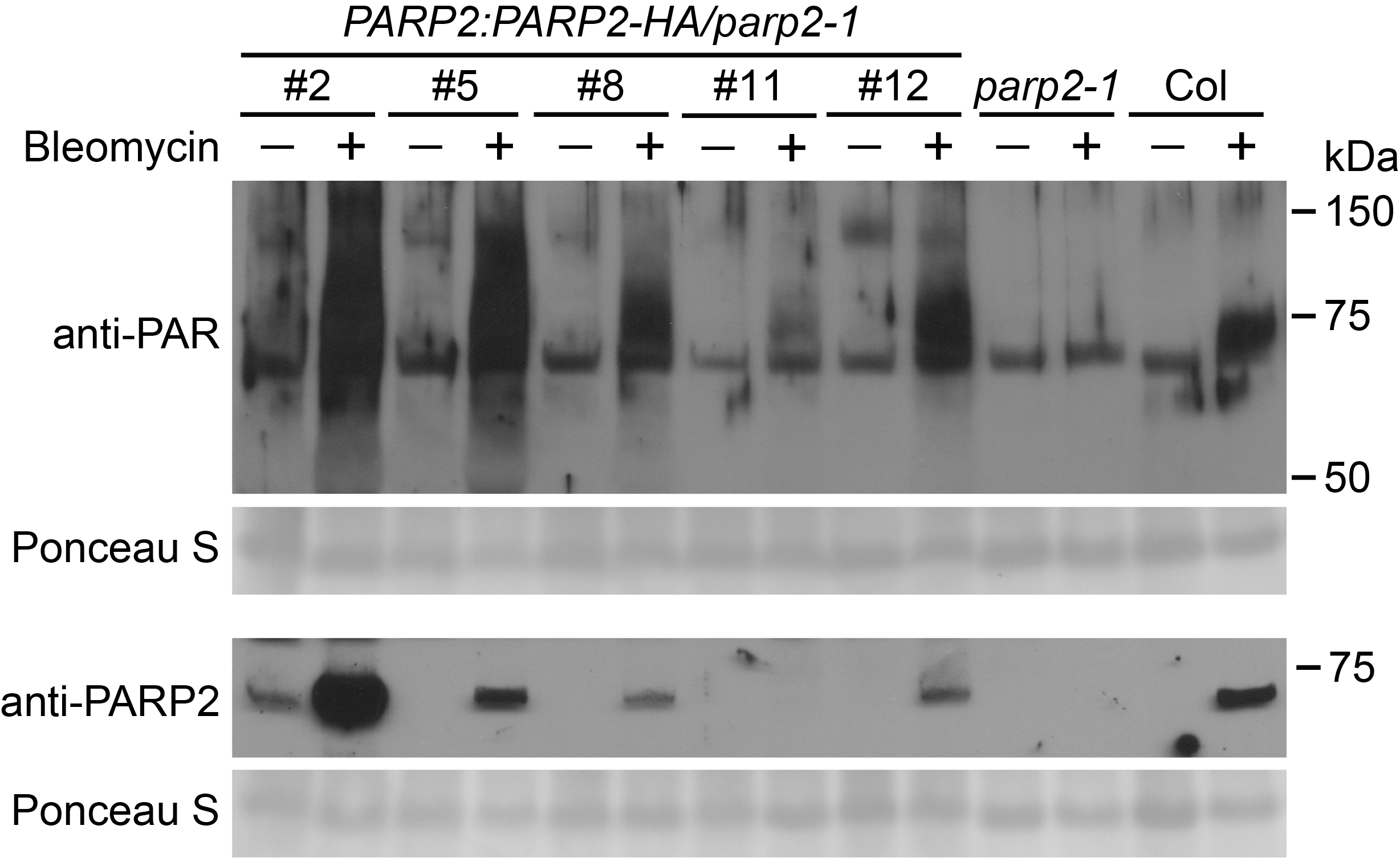

Supplement: S6 Fig — PARP2:PARP2-HA was transformed into the parp2-1 background. Five independent T2 lines, as well as parp2-1 and wild-type Col-0 plants included as negative and positive controls, were treated with 2.5 μg/ml of bleomycin for 18 h. Total proteins were extracted, separated by SDS-PAGE and detected with anti-poly(ADP-ribose) or anti-PARP2 antibody as indicated. Equivalent loading of lanes was verified using Ponceau S stain. (TIF) [file pgen.1005200.s006.tif]

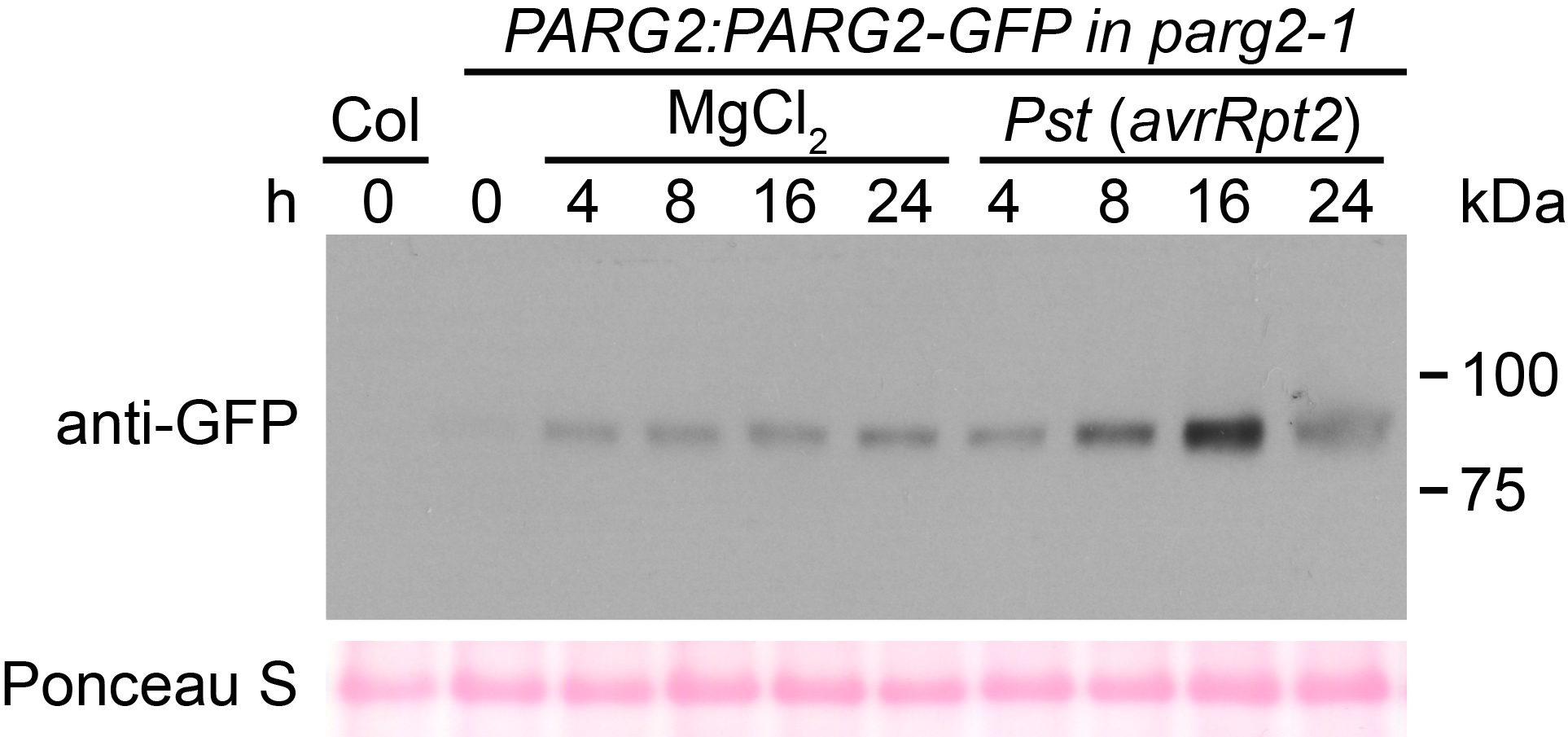

Supplement: S7 Fig — Five-week-old Arabidopsis parg2-1 mutant plants carrying PARG2:PARG2-GFP (2 kb of PARG2 promoter, PARG2 coding sequence fused to C-terminal GFP and nos terminator) were infiltrated with 10 mM MgCl2, or Pst DC3000(avrRpt2) at a concentration of 1×10^7^ cfu/ml in 10 mM MgCl2. Proteins were extracted at the indicated times, separated by SDS-PAGE and detected with anti-GFP antibody. Equivalent loading of lanes was verified using Ponceau S stain. Hypersensitive response-associated cell death is present in Arabidopsis Col-0 leaf tissues 24 h after infection by Pst avrRpt2. (TIF) [file pgen.1005200.s007.tif]

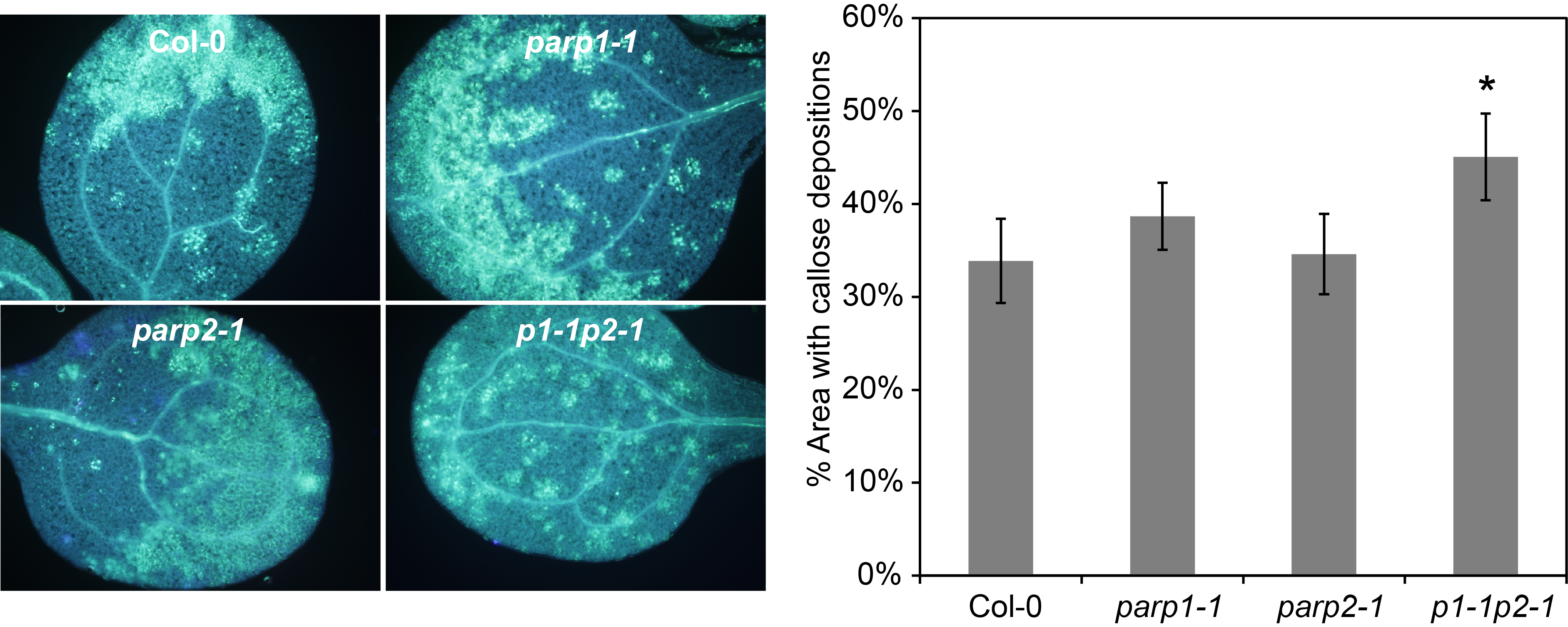

Supplement: S8 Fig — Seedlings exposed to 1 μM flg22 for 24 h were fixed and callose deposits were detected using aniline blue staining and quantified by ImageJ software. * indicates significant difference from Col-0 across the three experiments (ANOVA, Tukey pairwise comparisons, P < 0.05). (TIF) [file pgen.1005200.s008.tif]

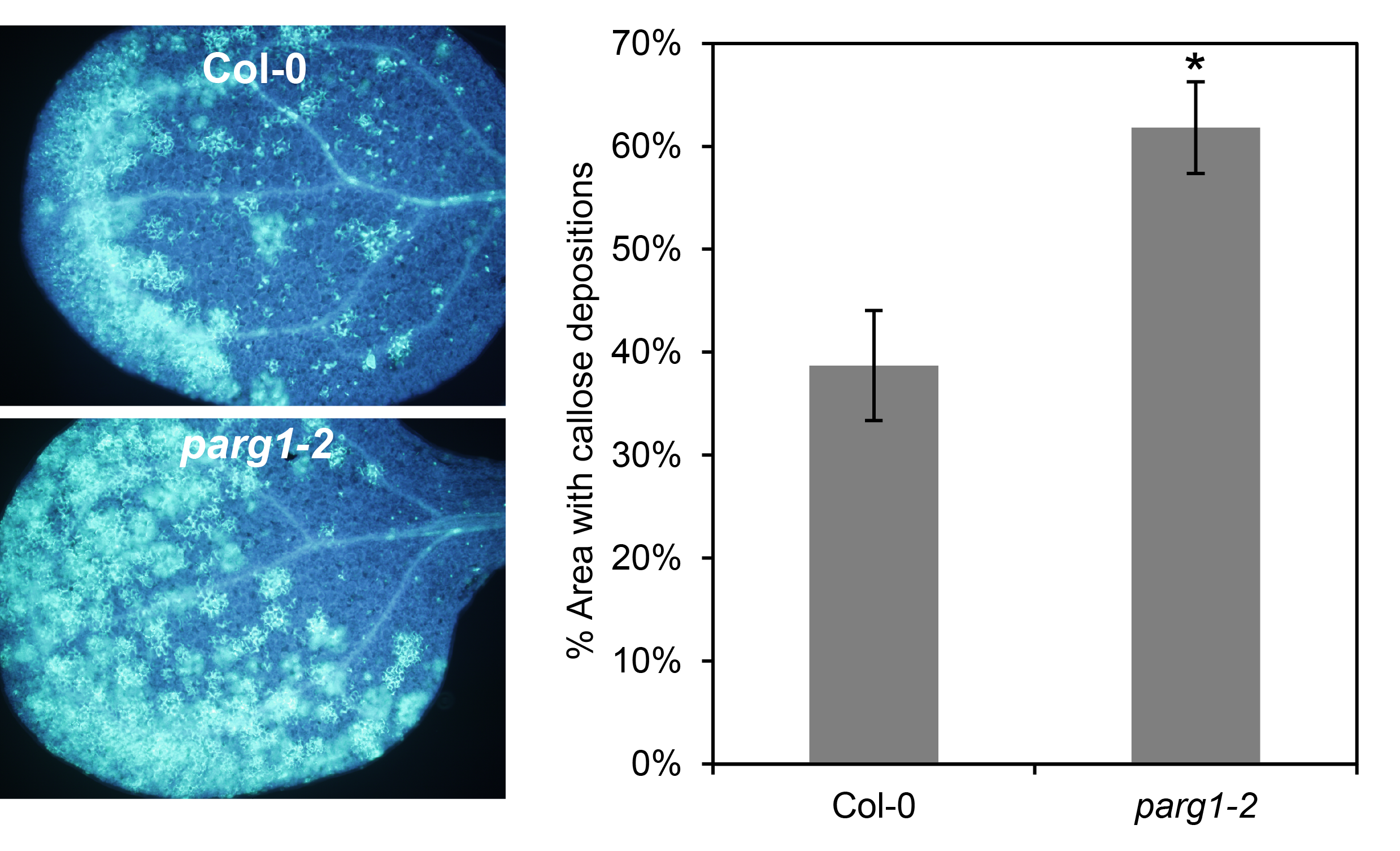

Supplement: S9 Fig — Seedlings exposed to 1 μM flg22 for 24 h were fixed and callose deposits were detected using aniline blue staining and quantified by ImageJ software. * indicates significant difference from Col-0 across the three experiments (ANOVA, Tukey pairwise comparisons, P < 0.05). (TIF) [file pgen.1005200.s009.tif]
